# Inhalable Mucociliary-On-Chip System Revealing Pulmonary Clearance Dynamics in Nanodrug Delivery

**DOI:** 10.1021/acsnano.4c11693

**Published:** 2025-01-07

**Authors:** Ko-Chih Lin, Hsuan-Yu Lin, Chuan-Yi Yang, Ying-Ling Chu, Ren-Hao Xie, Cheng-Ming Wang, Yun-Long Tseng, He−Ru Chen, Johnson H. Y. Chung, Jia-Wei Yang, Guan-Yu Chen

**Affiliations:** † Department of Electrical and Computer Engineering, National Yang Ming Chiao Tung University, Hsinchu 30010, Taiwan; ‡ Institute of Biomedical Engineering, College of Electrical and Computer Engineering, National Yang Ming Chiao Tung University, Hsinchu 30010, Taiwan; § Taiwan Liposome Company, Ltd, Taipei 11503, Taiwan; ∥ Intelligent Polymer Research Institute, AIIM Facility, University of Wollongong, Wollongong NSW 2500, Australia; ⊥ Anivance AI Corporation, Zhubei City, Hsinchu County 302058, Taiwan; # Department of Biological Science and Technology, College of Biological Science and Technology, National Yang Ming Chiao Tung University, Hsinchu 30010, Taiwan; ¶ Center for Intelligent Drug Systems and Smart Bio-devices (IDS^2^B), National Yang Ming Chiao Tung University, Hsinchu 300093, Taiwan

**Keywords:** microphysiological systems, breathing patterns, horizontal shear stress, mucosal barrier, inhaled nanodrug delivery, nanoparticle penetration, in vitro drug release

## Abstract

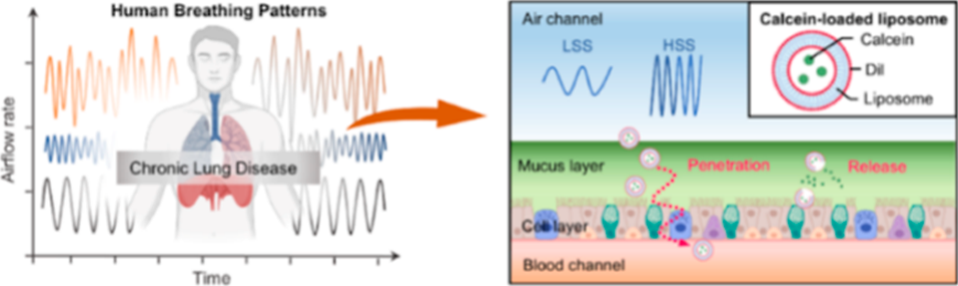

The development of a inhaled nanodrug delivery assessment platform is crucial for advancing treatments for chronic lung diseases. Traditional in vitro models and commercial aerosol systems fail to accurately simulate the complex human respiratory patterns and mucosal barriers. To address this, we have developed the breathing mucociliary-on-a-chip (BMC) platform, which replicates mucociliary clearance and respiratory dynamics in vitro. This platform allows for precise analysis of drug deposition and penetration, providing critical insights into how liposomes and other nanocarriers interact with lung tissues under various airflow conditions. Our results reveal that liposomes penetrate deeper into the cellular layer under high shear stress, with both static and dynamic airflows distinctly affecting their drug release rates. The BMC platform integrates dynamic inhalation systems with mucociliary clearance functionality, enabling a comprehensive evaluation of drug delivery efficacy. This approach highlights the importance of airflow dynamics in optimizing inhalable nanodrug delivery systems, improving nanocarrier design, and tailoring drug dosages and release strategies. The BMC platform represents a significant advancement in the field of inhaled nanodrug delivery, offering a more accurate and reliable method for assessing the performance of therapies. By providing a detailed understanding of drug interactions with lung tissues, this platform supports the development of personalized inhaled therapies and offers promising strategies for treating pulmonary diseases and advancing nanodrug development.

## Introduction

Patients with chronic lung diseases, such as COPD and asthma, often rely on inhaled medications. These conditions, often resulting in altered respiratory airflow rates and frequencies due to lung structure changes, can significantly affect the effectiveness of nanodrug delivery.^1^ Traditional research on inhaled medications tends to overlook the role of abnormal breathing patterns in influencing lung biology, a critical factor for effective medication delivery to specific lung regions and through the mucosal barrier.^2^ A thorough understanding of the impact of breathing patterns and biological mechanisms on nanodrug delivery processes is critical for enhancing the effectiveness of inhaled medications. Furthermore, there’s a noticeable gap in knowledge regarding the interaction of inhaled nanodrugs with the lung surface during their delivery. A primary challenge in developing inhaled medications is preserving their integrity while bypassing physiological barriers like mucus secretion and mucociliary clearance (MCC).^3^ Therefore, gaining insights into nanoparticle behavior within the lungs is essential for pharmacologists to create more effective inhaled nanomedicines.

Mucociliary clearance (MCC) is a principal mechanism for both the deposition and removal of foreign substances in the human airways, involving the mucus layer, periciliary layer (PCL), and respiratory airflow.^4,5^ The mucus layer captures and removes foreign particles through ciliary movement, with its viscosity being crucial for efficiency.^6^ The PCL supports this movement and influences clearance based on ciliary density and orientation.^7^ Moreover, altered ciliary function—often a result of airway damage—makes airflow the primary mechanism for mucus clearance, involving both gas-mucus transport and coughing.^8^ Despite the complexity of the lung’s structure and the unique air–liquid interface microenvironment, few experimental methods can effectively measure the dynamics of inhaled nanomedicines during airway mucus clearance with physiological respiratory airflow patterns at true scale. This limitation is due to traditional in vitro lower airway models’ inability to control both respiratory airflow and MCC parameters concurrently. Therefore, an in vitro model that can simulate the interaction between airflow mechanical stress and MCC is vital to explore the fate and permeability of inhaled drugs in lung tissue effectively.

The human lung-on-chip (LoC) model, leveraging in vitro microfluidic culture technology, holds potential for replicating the dynamics of human lung airway regions, like small airways and alveoli. This capability is valuable for studying the delivery of inhaled substances within the lung’s complex microenvironment.^9^ However, while many LoC models focus on cyclic stretching as the primary mechanical stress, few address the effects of shear stress induced by human respiratory airflow.^10^ In the airways, varying airflow rates across different regions result in differential shear stresses and mechanobiological stimulation to the lung epithelial cells. This airflow is essential for regulating mucociliary clearance and sustaining the integrity of the epithelial barrier, thereby significantly influencing the airway’s microenvironment.^11^ In studies of inhaled drug delivery, the efficacy of aerosolized drugs is significantly influenced by respiratory airflow and lung physiology.^12^ Although commercially available aerosol exposure systems simulate aerosol inhalation, they fail to accurately replicate the human respiratory profile for inhaled drugs. Additionally, the absence of a real-time monitoring system impedes precise observation of aerosol behavior in the human airways.^13^ Thus, there’s an urgent need to develop an in vitro platform that allows cells to experience mechanical stimulation mimicking the physiological conditions of human pulmonary respiration, enhancing our understanding of the fate of inhaled drugs in pulmonary airway tissue.

**Figure 1:**
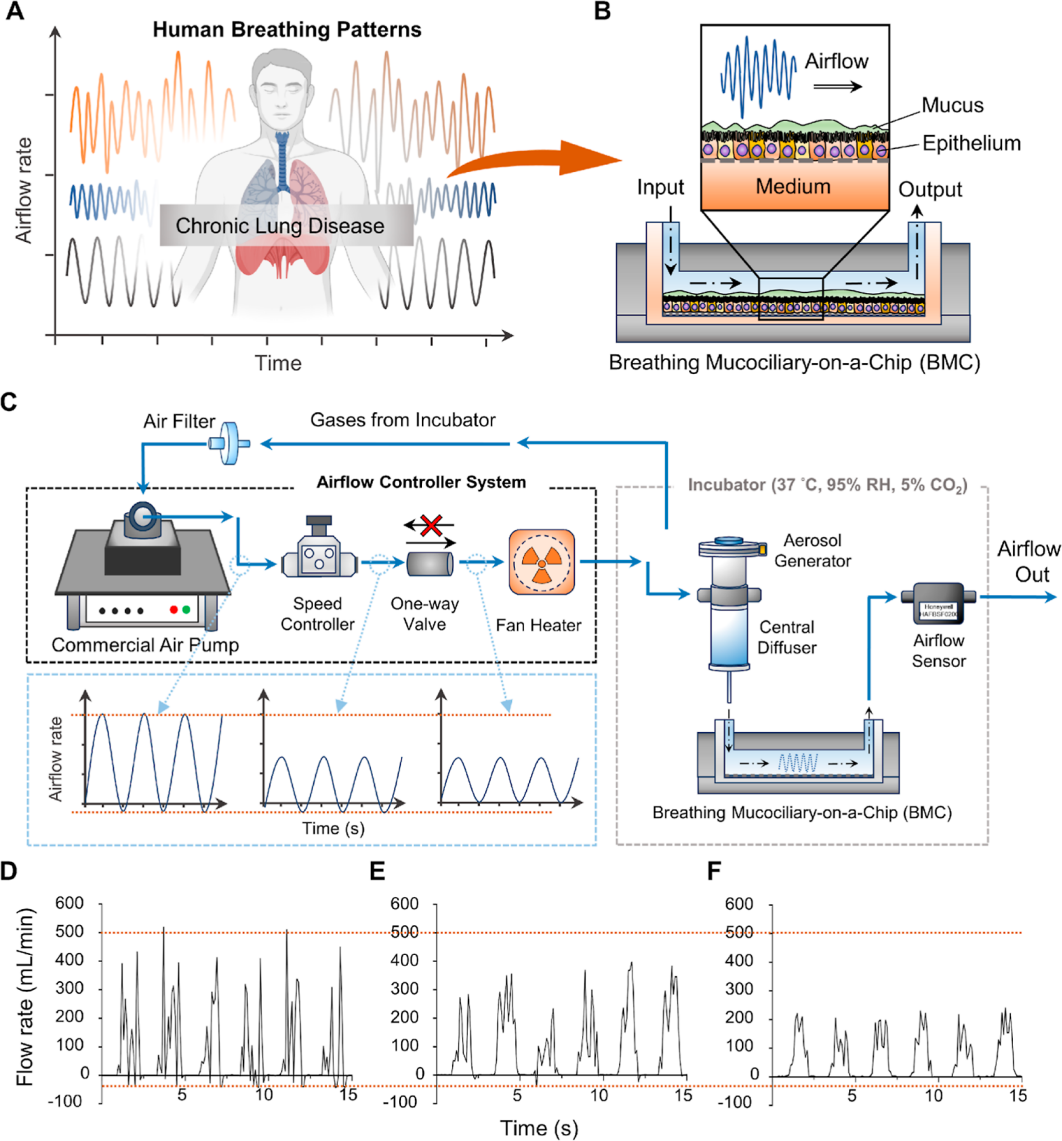
Configuration of BMC platform for establishing airflow patterns. (A) Schematic diagram of changes in various breathing patterns over time in patients with chronic lung disease. (B) The BMC (schematic diagram of the previous study [^17^) consists of bilayer channels. Upper channel is for culturing airway epithelial cells and airflow exposure; Lower channel is for media perfusion. (C) Illustration of BMC platform setup consisting of an airflow controller system, aerosol generator, central diffuser, and the BMC. The black dashed box highlights the components of the airflow controller system, including the commercial air pump, speed controller, one-way valve, and fan heater. The light blue dashed box illustrates the schematic diagram of airflow control changes within each component of the system. The gray dashed box indicates the parts operated within a cell culture incubator. (D–F) Plots depicting airflow patterns within a microchannel, demonstrating changes as the commercial air pump cycles through different settings of the airflow controller system across various flow rates.

To study this, we have significantly advanced with the creation of the breathing mucociliary-on-a-chip (BMC) platform. This sophisticated lung chip system effectively simulates the mucociliary clearance microenvironment and accurately replicates human respiratory dynamics in vitro (Figure 1A,B). Building upon our existing lung-on-a-chip device, which was limited to studying particulate matter penetration,^14^ our enhanced platform is now equipped to assess aerosol delivery and the effects of inhaled drugs. Featuring a biomimetic respiratory robot integrated with a human lung small airway chip, the platform facilitates a comprehensive investigation of various breathing patterns. This configuration allows for real-time observation of dynamic processes, such as mucus clearance and ciliary motion, under different respiratory conditions, achieved through a high-resolution imaging system. Additionally, our research delves into the penetration of nanoparticles, specifically liposomes, within the lung microenvironment on the chip. This offers pivotal insights into the influence of dynamic shear stresses, typical during particle inhalation on lung delivery. These significant improvements in our research methodology enhance our understanding of the interplay between breathing patterns, the mucosal barrier, and drug penetration, marking a step forward in creating a more dependable preclinical in vitro model for evaluating inhaled nanomedicines.

## Results

### Design and Characterization of the BMC Platform

In developing the BMC platform, our primary objective was to achieve precise airflow control and replicate human respiratory rates on the chip. Since the simulation of human respiration study indicated that the respiration pattern is correlated with the airflow rate and frequency,^15^ we focused on these elements as they are critical for determining the delivery dynamics of inhaled substances. Therefore, we divided the platform into three systems to do the integration of the lung chip, airflow controller system, and aerosol generator system to simulate the breathing state of human inhalation of foreign substances (Figure 1C). Building on our previously proposed PC chip,^14^ we have developed a design that includes two parallel microfluidic channels separated by a PET porous membrane. The upper channel is designed to create a microenvironment resembling human respiratory airflow for simulating the inhalation of particles. Simultaneously, the lower chamber mimics the microenvironment of human microvasculature.

Then, to accurately control the microairflow on the chip, thereby simulating the lung environment and specifically targeting the small airway area with shear stress under 10 dyn/cm^2^,^16^ we implemented a 2-fold strategy. First, a microairflow speed controller was added to the commercial air pump’s output, which reduces the airflow rate. This adjustment is crucial for replicating the delicate conditions of lung airways. Second, addressing the issue of airflow backflow—an inherent problem with the operation of the commercial air pump that leads to unstable reverse airflow—we installed an airflow one-way valve at the rear of the speed controller. This valve is essential in preventing reverse airflow, thus miniaturizing the flow rate and eliminating backflow, creating a half sine wave flow rate that mimics breathing. This feature is vital to ensure a stable direction of airflow in the system. Additionally, its precise regulation of microairflow is imperative for simulating the shear stress and stable half sine-wave changes of microairflow observed in human lungs’ small airways. Before entering the chip, airflow is drawn from the incubator, driven by a diaphragm pump, and heated to a constant temperature of 37 °C using a fan heater. It is then adjusted to maintain a relative humidity level typical of the lower respiratory tract, close to 100%. This regulation is essential for preserving cell viability by preventing the adverse effects of temperature and humidity fluctuations in cell culture. To ensure a seamless connection between the aerosol generator and the chip, we introduced a central diffuser that maintains an airtight system for smooth aerosol transfer into the microfluidic channel. This setup effectively simulates aerosol delivery in small human airways. To monitor detailed airflow behavior in the chip channel, we equipped the system with a real-time microairflow sensing system at the end of the microfluidic channel. This system is crucial for tracking instantaneous airflow changes within the chip. Sensor data (Figure 1D–F) reveals a significant reduction in airflow velocity postspeed controller, yet indicating persisting instability. The incorporation of a one-way valve not only further reduces the airflow but also ensures it remains stable and unidirectional, as confirmed by the absence of *y* < 0 values, indicating no backflow. Using programmable software, we precisely controlled the output airflow’s size and waveform, allowing for the customized simulation of various respiratory parameters such as airflow magnitude and frequency. This customization is essential for replicating diverse physiological conditions in our experiments. Additionally, integrating a one-way valve optimized the system by reducing airflow velocity and enhancing its stability and directionality. Sensor data confirmed this approach’s effectiveness, showing no negative values, thereby indicating the successful elimination of backflow. These enhancements provide a robust platform for accurately simulating human respiratory mechanics, marking a significant advancement over our previous design and offering more precise simulations of airflow dynamics and their effects on inhaled nanodrug delivery systems.

**Figure 2:**
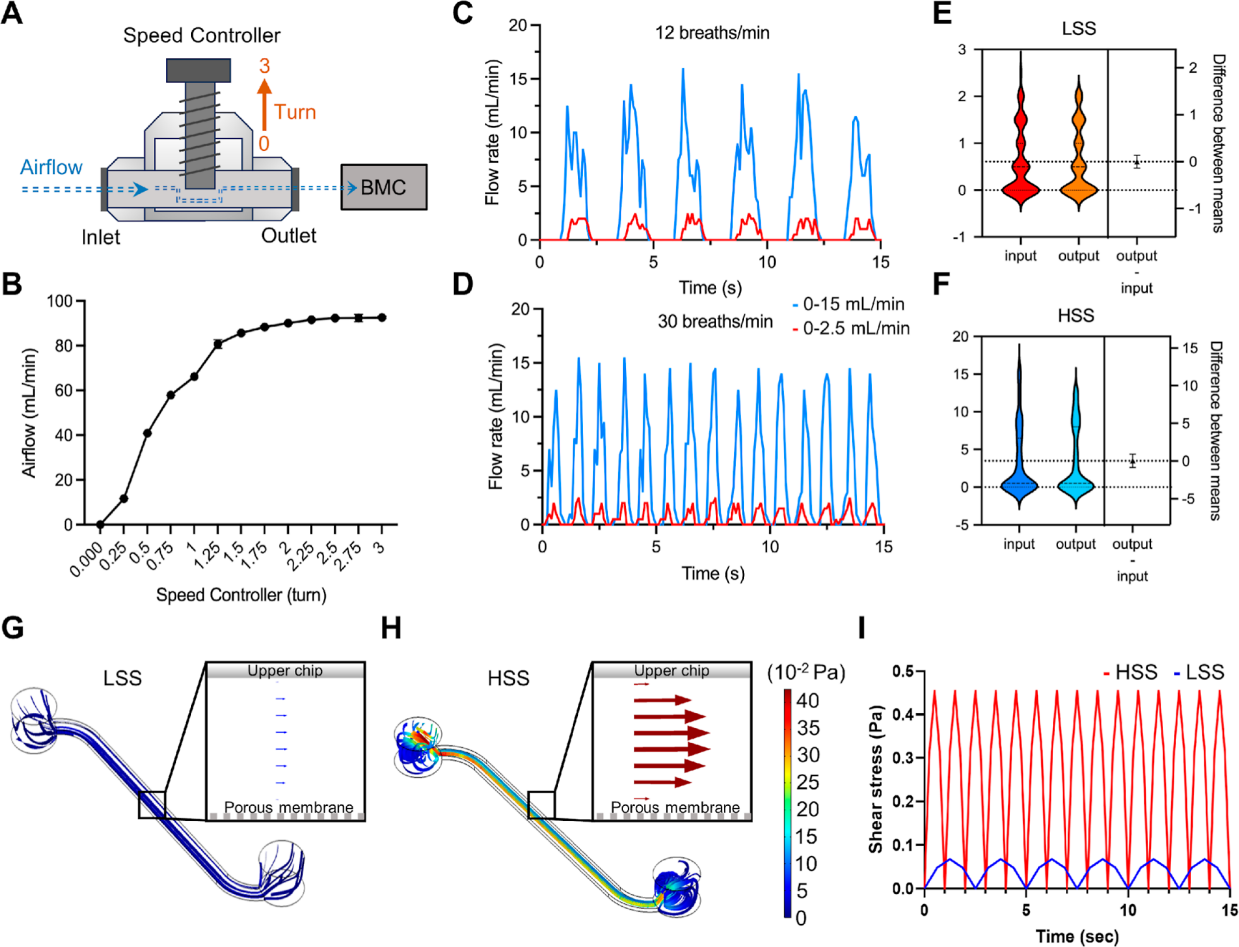
Characterization of the BMC platform for breathing pattern simulation. (A) Schematic diagram of the speed controller detailing its working mechanism. The middle shaft rotates between 0 and 3 turns to adjust airflow rates from the inlet to the outlet. (B) Graph depicting the relationship between the number of turns on the speed controller and the corresponding airflow rates. (C,D) Plots representing different respiratory patterns for flow rates of 0–15 mL/min (blue) and 0–2.5 mL/min (red) at breathing rates of 12 breaths/min and 30 breaths/min, respectively. (E,F) Violin plots illustrating the differences between mean airflow input and output under LSS and HSS conditions. (G,H) COMSOL simulations of airflow velocity within the microchannels under LSS and HSS conditions, demonstrating the airflow paths and shear stress distribution. (I) Shear stress profiles over time for HSS (red) and LSS (blue) conditions, highlighting variations in shear stress across different respiratory cycles.

Next, to validate airflow customization on the BMC platform, we first determined the airflow range into the chip. Controlled by a speed controller with three adjustable valve turns, the airflow increases with each turn. Sensor data showed an airflow range from 0 to 94 mL/min, equivalent to 39.5 dyn/cm^2^. This range suggests that our system can replicate most human lung respiratory shear stresses within the chip (Figure 2A,B).^17^ Our research focuses on the small airway regions of the lungs, which are typically subjected to shear stresses between 1 and 5 dyn/cm^2^.^16^ To enhance the relevance of our studies, we have devised several breathing patterns that accurately simulate these shear stresses. Specifically, we selected two respiratory patterns for detailed analysis: a flow rate of 2.5 mL/min at 12 breaths/min (a 5 s breath cycle) and a flow rate of 15 mL/min at 30 breaths/min (a 2 s breath cycle), corresponding to shear stresses of 1.16 and 7.44 dyn/cm^2^, respectively. These represent low shear stress (LSS) and high shear stress (HSS) conditions, emulating normal breathing and more extreme conditions like hyperventilation seen in chronic lung diseases (Figure 2C,D).^18^ Although human pulmonary situations involve reciprocal air flow-in and flow-out during the respiratory cycle, our study simplifies this process to create stable airflow in the microchannel. We focus on how different shear stresses from human breathing patterns affect the delivery of foreign substances in the airways. To this end, we have incorporated a one-way valve to transform the negative half-wave (*y* < 0) of the human respiratory sine wave into a positive value. Consequently, each respiratory cycle in the microchannel produces two positive sine waves. This modification ensures that the system’s frequency and shear stress align with those of a full human respiratory cycle, including exhalation phases. Additionally, our results demonstrate consistent airflow at the chip’s inlet and outlet under both low and high airflow conditions, confirming the system’s accuracy and stability in delivering airflow to the chip (Figure 2E,F).

To show the physiological relevance of respiratory patterns produced by our BMC platform to human lungs, we employed COMSOL Multiphysics software for dynamic 3D simulation of the above two breathing patterns within the chip. These simulations focused on the two specified breathing patterns within the chip, analyzing airflow magnitude and frequency. The results showed the uniform respiratory airflow distribution and stable delivery in the microchannels. Notably, our simulations demonstrated that airflow near the chip’s walls formed a parabolic velocity curve, leading to reduced velocities in these regions, a pattern that aligns with established fluid dynamics principles (Figures 2G,H, and S1).^19^ Additionally, the model registered shear stresses of 0.08 Pa in low shear conditions and 0.45 Pa in high shear conditions. These values are consistent with the shear stress ranges typically observed in human small airways, confirming both the physiological accuracy and the relevance of our model (Figure 2I).^16^ Thus, our chip not only replicates the shear stress experienced during human breathing but also confirms that changes in the size and frequency of respiratory airflow can lead to corresponding shifts in shear stress within the chip.

### Influence of Respiratory Patterns on Aerosol Delivery and Deposition in BMC Platform

**Figure 3:**
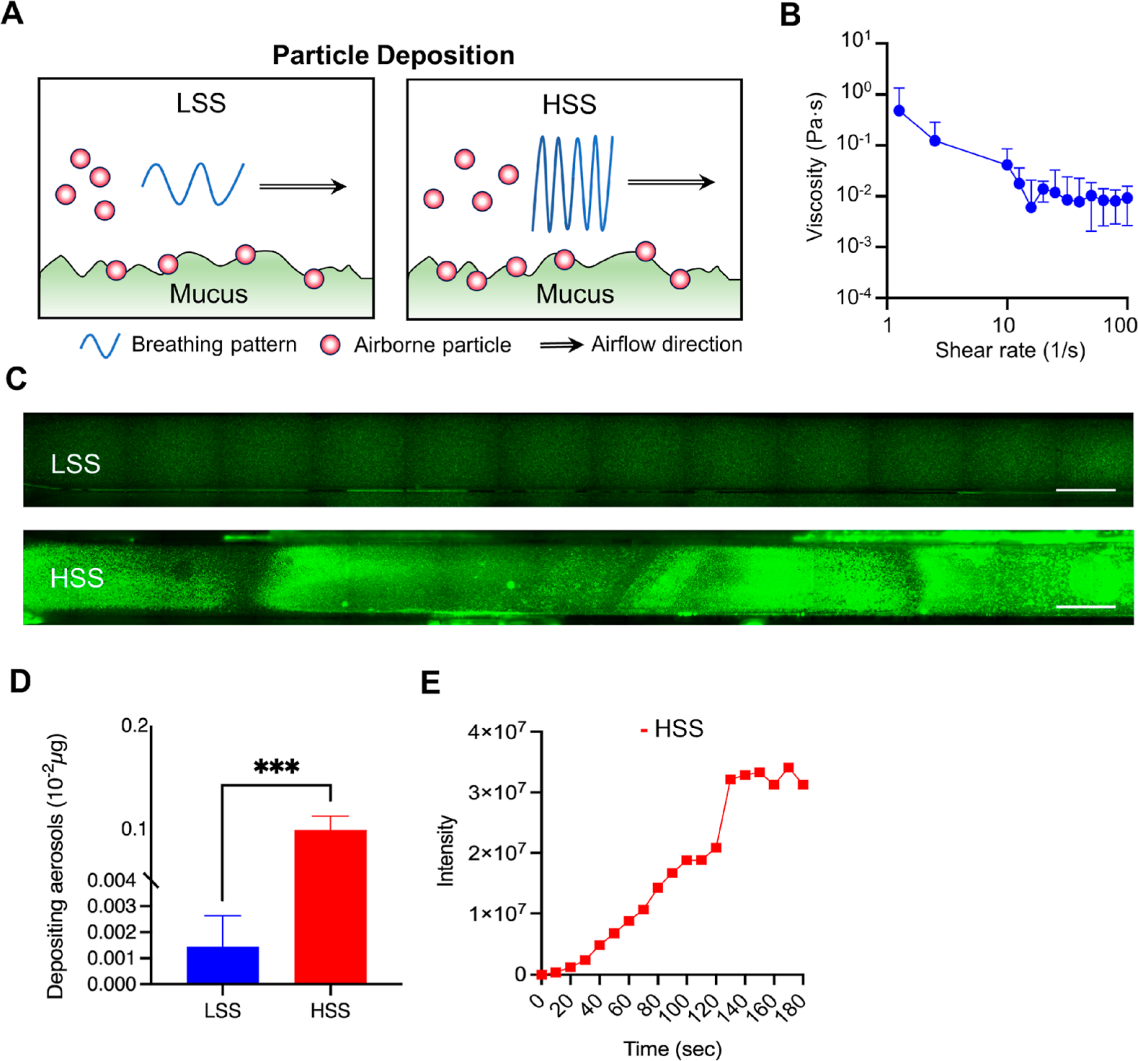
Particle deposition and mucus rheological properties under breathing patterns. (A) Schematic representation of particle deposition in the mucus layer under LSS and HSS conditions. The airflow patterns depict the differences in particle behavior between the two conditions. (B) Rheometer results showing the relationship between shear rate and viscosity of artificial mucus. Viscosity measurements decrease with increasing shear rate, indicating the shear-thinning behavior of mucus. (C) Fluorescence microscopy images of particle deposition in the mucus layer under LSS and HSS conditions. The images illustrate higher particle deposition under HSS. Scale bars: 1000 μm. (D) Quantification of depositing aerosols under LSS and HSS conditions. The bar graph shows significantly higher aerosol deposition under HSS. Statistics were performed using 1-way ANOVA with multiple comparisons; ***P < 0.001. (E) Intensity of deposited particles over time under HSS conditions, demonstrating an increase in particle deposition until it reaches a plateau. The deposition concentration appears to reach saturation over time under the influence of respiratory airflow.

After establishing various respiratory patterns in the chip, we initially focused on how these patterns influence aerosol delivery and deposition. We used a fluorescent saline solution as the aerosol to observe and quantify both its deposition and distribution in the lung chip. This helped us easily analyze the interplay between aerosol behavior and respiratory patterns. The delivery of saline solution was carried out under both LSS and HSS conditions. We then used high content screening (HCS) to observe and quantify the fluorescence intensity deposited on the PET membrane of chip, further exploring the effect of respiratory patterns on aerosol deposition (Figure 3A). Considering that mucus is the primary biological barrier on human airway surfaces, its viscoelastic properties play a crucial role in the deposition and transport of inhaled substances.^20^ Previous studies have utilized collagen to simulate airway mucus in in vitro lung models, focusing on particle deposition dynamics.^21^ However, mucins are pivotal in dictating mucus behavior, significantly influencing its viscoelasticity and, consequently, the deposition and distribution of inhaled substances.^18^ In this study, we first developed artificial mucus using 4% MUC5AC and 4% MUC5B mucins, cross-linked with 4-arm PEG-thiol to simulate the disulfide bonds of natural respiratory tract mucus.^22^ This allows for a precise evaluation of the interactions between respiratory patterns, mucus viscosity, and aerosol deposition. Additionally, since mucus is a non-Newtonian fluid that displaying a decrease in viscosity with increasing shear rate, exhibiting a “shear thinning” phenomenon.^23^ Our rheometer tests showed a consistent decrease in the viscosity of artificial mucus as shear stress from airflow increased (Figure 3B). This behavior mirrors human respiration and mucus interaction and is essential for understanding particle deposition.

We then examined the distribution and deposition of fluorescent saline in the chip under different respiratory patterns. Both fluorescence imaging and quantitative analyses revealed that fluorescence intensity was significantly higher under the HSS condition compared to the LSS condition, suggesting increased deposition rates with higher airflow (Figure 3C,D).^24^ In addition, we explored how concentration affects aerosol distribution and deposition in our system, using 50, 100, and 400 μg/mL as test concentrations. At 50 and 100 μg/mL, aerosol deposition was notably higher at the anterior and posterior ends of the chip than in the middle, with increased airflow contributing to this pattern. In contrast, at 400 μg/mL, deposition was primarily concentrated toward the chip’s posterior end (Figure S2). These observations are consistent with Nof et al.’s 2022 study, which found that airflow in microchannels causes turbulent flow in areas like nasal pockets and bronchial bifurcations, thus increasing aerosol inertial impaction and deposition.^25^ Our chip’s design, which includes curved channels at the front and back to connect to the air exposure pipeline, replicates these anatomical features, leading to enhanced particle wall impaction in these regions.^26^ The distinct deposition patterns at 400 μg/mL, compared to lower concentrations, could be due to the higher fluorescent particle concentration leading to increased variability in exposure, deposition, and distribution. Nevertheless, we did not investigate these patterns further, as such high concentrations are not typical in human airways and were not the focus of our study. Finally, we monitored the deposition changes of fluorescent particles in the chip following continuous exposure. In HSS condition, particle fluorescence intensity was tracked over time to indicate deposition rate. We observed an increase particle fluorescence intensity with each respiratory cycle, eventually reaching the steady state after 2 min of exposure. This led to a stable concentration distribution in the microchannels (Figure 3E). Our results confirm the system’s ability to accurately simulate the delivery and deposition of inhaled substances under varying respiratory patterns, highlighting their substantial impact on particle distribution within the airway mucus environment. Furthermore, our findings demonstrate that the system effectively replicates the influence of respiratory cycles on particle deposition, validating its efficacy in modeling human conditions in pulmonary therapy.

### Impact of Respiratory Patterns on MCC in BMC Platform

**Figure 4:**
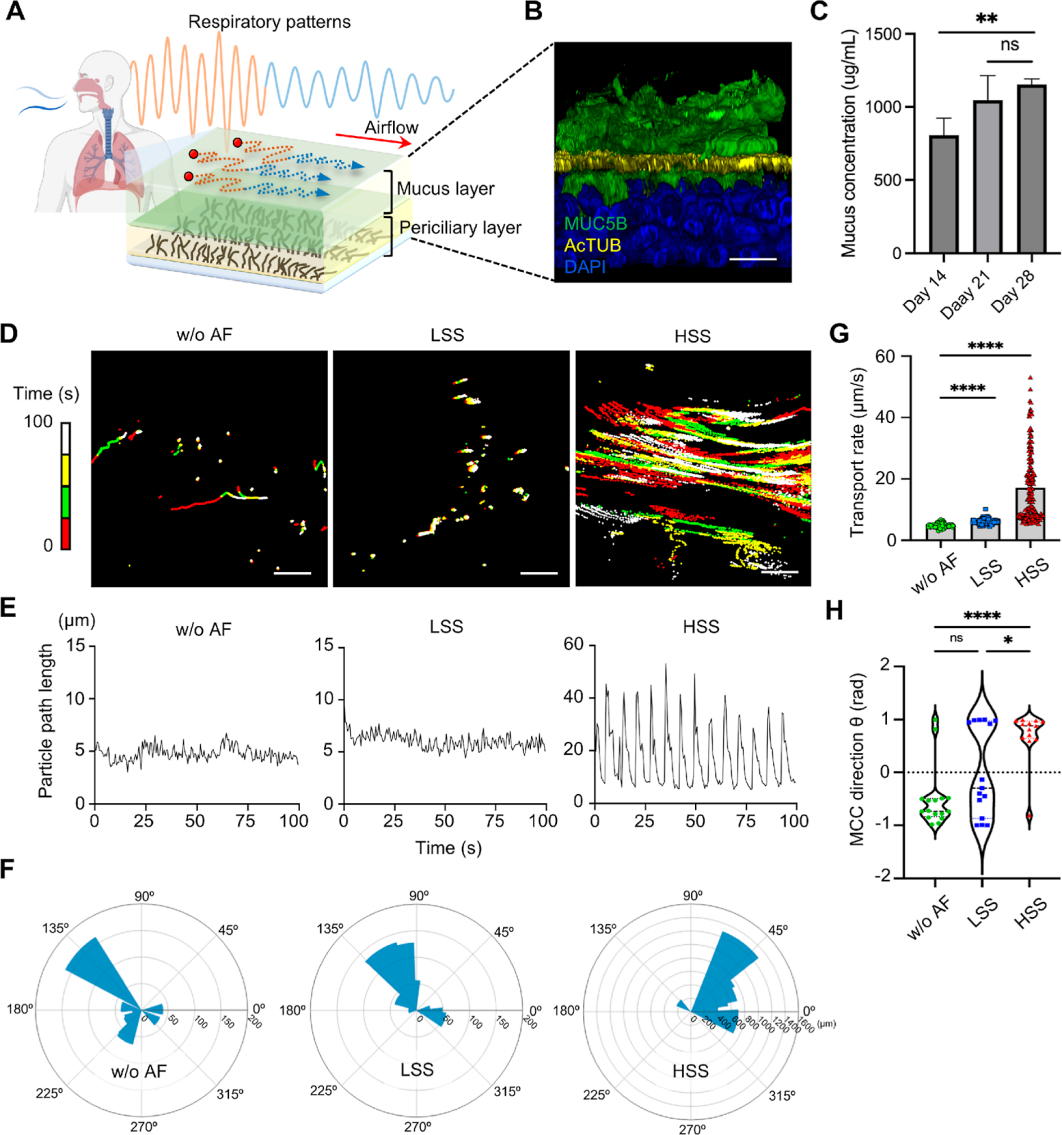
Effects of respiratory patterns on mucociliary clearance (MCC). (A) Schematic representation of the BMC platform, illustrating the influence of different respiratory patterns on the mucus and periciliary layers. (B) Immunofluorescence image showing the mucus layer stained for MUC5B (green), cilia with Ac-tubulin (yellow), and cell nuclei with DAPI (blue). Scale bar: 20 μm. (C) Bar graph showing the mucus concentration (μg/mL) in the BMC on day 14, 21, and 28. Statistics were performed using 1-way ANOVA with multiple comparisons; **P < 0.01, ns indicates nonsignificant differences. (D) Particle trajectories in the mucus layer, recorded every 25 s in various colors for clarity, under three conditions: w/o AF, LSS, and HSS. Scale bars: 100 μm. See also Video S1. (E) Graphs depicting the particle path length over time for each condition, demonstrating increased path length under HSS conditions. (F) Polar plots showing the directionality of particle movement under each condition. (G) Bar graph illustrating the transport rate of particles (μm/s) under different conditions. Statistics were performed using 1-way ANOVA with multiple comparisons; ****P < 0.0001. (H) Violin plot showing the MCC direction (θ in radians) under various conditions. Statistics were performed using 1-way ANOVA with multiple comparisons; **P < 0.01, *P < 0.05, ns indicates nonsignificant differences.

Our research explores how respiratory patterns affect particle deposition on our BMC platform. Next, we cultured human small airway epithelial cells (HSAEC) on the chip under an air–liquid interface for 21 days, allowing them to differentiate into pseudostratified epithelial tissue capable of mucociliary clearance. We then exposed these tissues to both LSS and HSS conditions to assess how different breathing patterns influence the mucus and ciliated layer (PCL). Consequently, this exposure provided insights into the effects on MCC and the efficiency of particle clearance in the airways (Figure 4A). We validated the integrity of the epithelial barrier using ZO-1 immunofluorescent staining, which confirmed the presence of a robust tight barrier essential for maintaining a pseudostratified structure.^27^ To evaluate the distribution and differentiation of ciliated cells, we employed AC-tubulin fluorescent staining, verifying their functional presence (Figure S3). Recognizing that changes in the mucus layer, including secretion volume or thickness, can affect MCC functionality due to lung diseases,^28^ we also measured the mucus layer thickness using MUC5B protein fluorescent reactions. This thickness varied from approximately 5 to 30 μm depending on the culture duration, aligning with in vivo findings.^29^ As a model for chronic lung disease conditions, we used a thicker mucus layer, approximately 20–30 μm by day 21, for our experiments (Figure 4B). To quantify mucus production, we utilized a sulfated-Glycosaminoglycans Assay Kit along with spectrophotometric analysis.^5^ We found that the cell mucus secretion reached maturity by the third week, showing no significant differences between weeks three and four (Figure 4C). Subsequent functional validation of these matured HSAEC included assessments of ciliary beating and MCC, enabling us to evaluate lung function across different respiratory patterns.

We investigated various factors affecting MCC, particularly focusing on mucus secretion and ciliary movement.^30^ To analyze MCC rates, we tracked the movement of fluorescent particles in the microfluidic chip under different respiratory patterns, recording particle trajectories every 25 s in various colors for clarity. The results indicated that in the no airflow (w/o AF) group, particle movement was more random compared to the LSS and HSS groups, which exhibited more uniform directions. Specifically, under HSS, particles showed intense back-and-forth movement, aligning over time with the airflow direction (Figure 4D and Video S1). In addition to visual analysis, we quantified the displacement per second (Figure 4E) and transport rates of particles. The average transport rates in the w/o AF, LSS, and HSS groups were 4.76, 6.04 and 17.11 μm/s, respectively (Figure 4G). The differences in particle movement and transport rates between the groups are notable; the LSS group’s speed was 1.27 times that of the w/o AF group, while the HSS group’s speed was 3.59 times faster. These findings illustrate how variations in airflow can significantly influence MCC, with the HSS group displaying the most efficient particle clearance, significantly higher than that of the LSS group. This suggests that the intensity of the airflow, mimicking more rigorous respiratory conditions such as hyperventilation, can enhance the clearance capabilities of the epithelial surface. While the w/o AF group did show some particle movement, the clearance rate was slightly divergent from typical in vivo reactions, highlighting the critical role of airflow in effective mucociliary clearance.^31^ These results highlight the significant impact of respiratory airflow on MCC and affirm that our model effectively simulates human-like MCC behavior, offering a physiologically relevant method for in vitro study of mucociliary clearance.

We also analyzed the directionality of particle movement. The results showed the leftward MCC direction in the w/o AF group, but introducing respiratory airflow changed this pattern (Figure 4F,H). We hypothesize that even small airflows increase both the average and instantaneous particle movement and can alter MCC direction, potentially overpowering ciliary movement. Under conditions of larger airflows, there was a notable shift in particle movement, suggesting the disruption of the mucus layer which facilitated substantial particle advancement, akin to mechanisms observed in cough-induced mucus clearance.^32^ Therefore, our BMC platform not only provides observations of more realistic mucus clearance but also simulates cough-like clearing scenarios. This model could offer diverse scenarios for studying drug movement behavior in future work. Furthermore, the results indicate that the CBF in our BMC closely resembles that found in human physiology, validating our model’s accuracy.^33^ Importantly, we observed no significant difference in CBF during airflow exposure (Figure S4), suggesting that the mucus layer shields the cilia from airflow effects, thereby maintaining the independence of ciliary movement from airflow dynamics.

### Implications of Horizontal Shear Stress on Liposome Behavior in the Mucosal Barrier and Correlation with In Vivo Data

**Figure 5:**
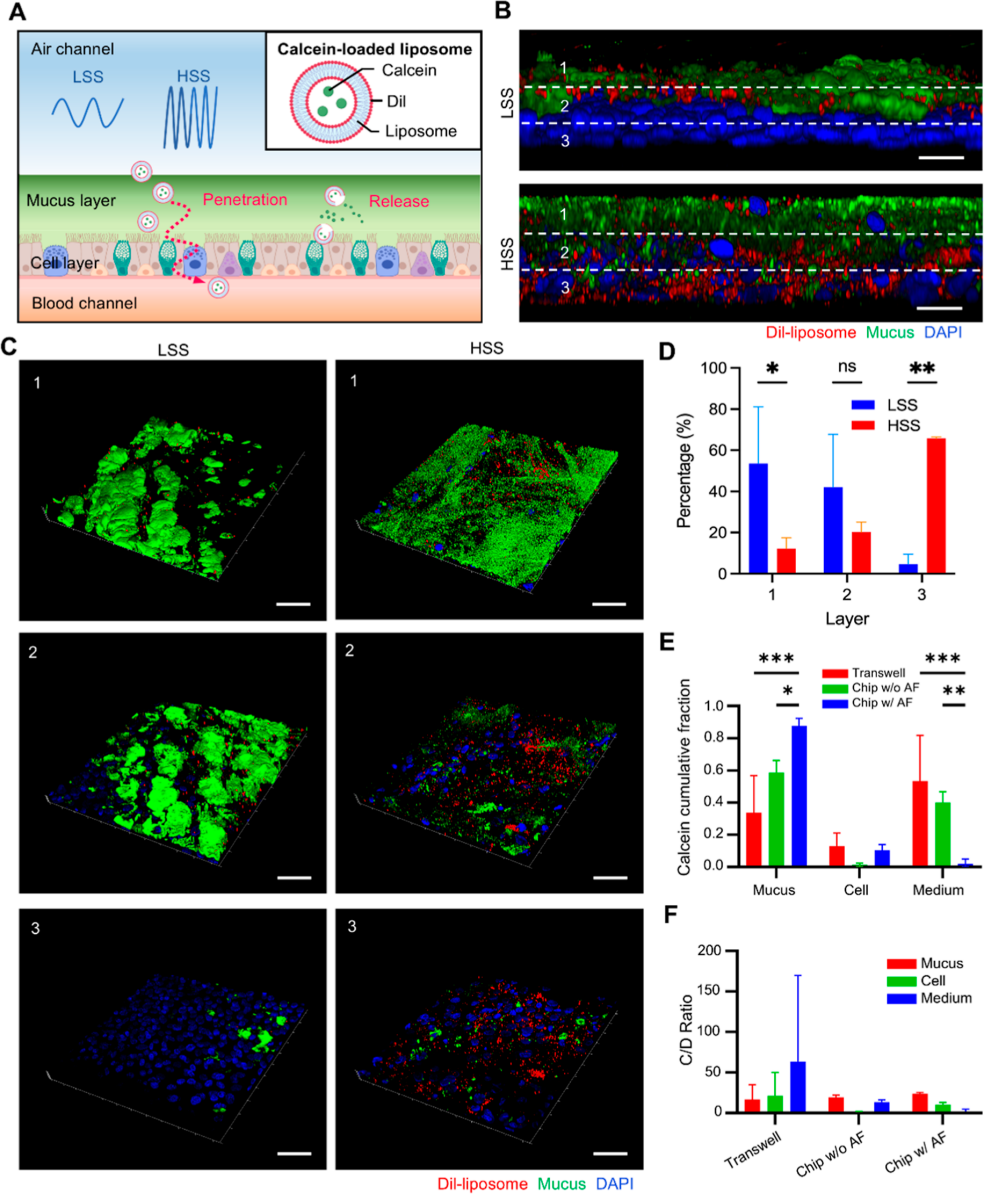
Penetration and release of calcein-loaded liposomes in the BMC platform under different breathing patterns. (A) Schematic illustration of calcein-loaded liposome behavior under LSS and HSS conditions. The diagram depicts the penetration and release process of the liposomes across the mucus and cell layers. (B) Confocal microscopy images showing the distribution of DiI-liposomes (red), mucus (green), and nuclei (blue) under LSS and HSS conditions. The layers are divided into three regions: (1) mucus, (2) mucus-cell interface, and (3) cell. Scale bars: 20 μm. (C) 3D confocal images of liposome penetration in the mucosal barrier under LSS and HSS conditions. The images represent the entire thickness of the mucosal barrier divided into three equal sections for standardized analysis: (1) upper section (primarily mucus), (2) middle section (mucus–cell interface), and (3) lower section (primarily cellular). This division is proportional to the total barrier thickness for each condition, allowing for consistent analysis across different shear stress conditions. Scale bars: 50 μm. (D) Quantification of liposome penetration across the three layers under LSS and HSS conditions. Statistics were performed using 1-way ANOVA with multiple comparisons; *P < 0.05, **P < 0.01, ns indicates nonsignificant differences. (E) Calcein cumulative fraction in the mucus, cell, and medium for transwell, chip w/o AF, and chip w/AF setups. Statistics were performed using 1-way ANOVA with multiple comparisons; *P < 0.05, ***P < 0.001. (F) Ratio of calcein to DiI (C/D) in the mucus, cell, and medium for transwell, chip w/o AF, and chip w/AF setups, indicating the release dynamics of calcein from the liposomes.

Our findings confirm that horizontal shear stress generated by respiratory airflow in human airways affects mucus movement along the airway surface. This may be critical for the pulmonary delivery of inhaled nanocarrier drugs in patients with chronic lung diseases. Therefore, we selected liposomes, widely used as inhaled nanocarriers, as experimental subjects to explore their behavior under shear stress at the airway epithelial tissue level. We specifically employed calcein-loaded liposomes to facilitate observation and study of drug penetration and release across various respiratory patterns. Since surface modifications of nanocarriers impact their deposition and penetration in the respiratory tract, we exposed two types with distinct surface properties but identical diameter (100 nm): hydrophilic carboxylate-modified FluoSpheres and hydrophobic DiI liposomes, to the BMC platform. The flow cytometry analysis verified the uniform particle size distribution of both types, each measuring approximately 100 nm in diameter (Figure S5). After depositing the drugs on the epithelial tissue surface, we subjected them to LSS and HSS conditions for 10–20 min. The tissues were subsequently fixed for confocal microscopy analysis, which allowed us to observe the penetration and release of nanoparticles under the varying shear stresses induced by different respiratory patterns (Figure 5A).

In our 3D fluorescence imaging side views, an interesting observation was that the majority of 100 nm carboxylate-modified FluoSpheres, under both LSS and HSS conditions, predominantly remained within the mucus layer with minimal cellular layer penetration (Figure S6). In contrast, DiI liposomes exhibited limited penetration in LSS but showed significantly higher cellular layer penetration under HSS (Figure 5B). To further quantify the liposomes’ penetration differences under varying shear stresses, we divided the side views of 3D fluorescence images into three equal sections, roughly corresponding to the mucus layer, the mucus–cell interface, and the cellular layer. We then measured the proportion of red fluorescence pixels in each section relative to the total, to gauge DiI liposome penetration depth along the *z*-axis (Figure 5C). In the LSS group, about 53.54% of DiI liposomes stayed in the first (mucus) layer, and 42.03% reached the second layer, but only 4.64% penetrated to the third layer. However, under HSS, about 65.73% of DiI liposomes were found in the third layer, with just 12.25% and 20.24% in the first and second layers, respectively (Figure 5D). A notable difference in DiI liposomes distribution between the LSS and HSS groups was observed in the first and third layers, confirming a deeper distribution tendency under HSS. These findings demonstrate that horizontal shear stress significantly influences liposome penetration, highlighting the importance of dynamic airflow in evaluating inhalable drug delivery.

Additionally, we explored the release rates of drugs from liposomes influenced by their hydrophilic or hydrophobic properties and polar surface area (PSA). To understand how horizontal shear stress affects drug release from liposomes in vitro, we employed DiI-calcein liposomes with calcein as a model drug. Calcein is known for its self-quenching properties at high concentrations,^34^ which allowed us to measure drug release by monitoring changes in fluorescence intensity. For comparative analysis, calcein concentration changes in the culture medium over 24 h were examined using three setups: traditional static drug release (transwell), dynamic chip with airflow (chip w/AF), and static chip without airflow (chip w/o AF). We quantified the cumulative distribution of calcein across the mucus layer, cell layer, and culture medium at the 24 h mark (Figure 5E,F). Our results revealed similar initial deposition rates of Dil-liposome in both the transwell and static chip setups (Figure S7). However, after 24 h of air exposure, significant differences in calcein penetration were observed. In static environments, calcein penetration was notably higher compared to the dynamic chip (chip w/AF), where penetration was only 1.97% (Figure 5E). This outcome correlates with prior animal studies showing that approximately 87.69% of calcein remained within the mucus layer.^35^ The calcein/DiI (C/D) ratio analysis indicated an enhanced drug release under dynamic conditions, suggesting that dynamic shear stress may amplify liposome drug release.^36^ The calcein C/D ratios in both transwell and chip w/o AF setups mirrored those observed in fresh DiI-calcein liposomes, yet release rates varied significantly. Notably, the transwell setup displayed a peak concentration (*C*_max_) 71.9 times greater than that in the chip w/AF, underscoring the stark differences in release rates between the setups. The dynamic chip demonstrated more pronounced changes in release rates, as detailed in Table S1. After 2 h, the cumulative release rate of calcein in the chip w/AF was significantly slower than in static groups. This sustained-release pattern continuously increased calcein concentration in the culture medium over 24 h, contrasting with the decreasing trends typically observed in vivo. These observations suggest that dynamic shear stress not only enhances initial drug release but also influences the long-term release profile of liposomal drugs.

Finally, to evaluate our BMC’s ability to predict in vitro-in vivo correlation (IVIVC), we employed noncompartmental analysis (NCA) to transform DiI-calcein liposome release data into area-under-curve (AUC) vs time plots (Figure S8A). This analysis revealed that static conditions, such as the transwell and chip without airflow, demonstrated linear AUC-time relationships. Conversely, the dynamic chip with airflow exhibited a steeper AUC increase initially, suggesting that airflow enhances drug penetration. We further validated these in vitro AUC findings against in vivo data from liposome animal trials using point-to-point level A IVIVC analysis. All in vitro models correlated strongly with in vivo outcomes,^37^ achieving *R*^2^ values greater than 0.9, with the transwell setup showing the highest correlation at 0.9936, indicating its excellent predictive relevance (Figure S8B and Table S1). Moreover, our platform demonstrated its effectiveness in simulating the impact of mucus composition on inhalable drug delivery, particularly under conditions that mimic cystic fibrosis. In such scenarios, where mucin levels are 5 to 10 times higher than in healthy individuals,^38^ we observed increased mucus thickness and viscosity, leading to small airway obstructions and reduced airflow shear stress. These alterations significantly affect drug delivery efficiency and nanomedicine penetration, underscoring the BMC platform’s utility in studying inhalable drug dynamics in pathologically relevant settings.

## Discussion

In this study, we developed a breathing mucociliary-on-chip (BMC) platform using air exposure systems and lung-on-chip technology to analyze the interaction of inhaled particles with the mucosal barrier. The design of the air exposure system allows for the engineering of controllable respiratory patterns that influence the mucus layer in the microchannels. This is facilitated by a microairflow speed controller and a one-way valve, which together adjust and stabilize airflow. In parallel, engineered lung epithelial tissues were evaluated for mucociliary clearance under controlled airflow conditions. The robustness of the BMC platform was validated by observing particle trajectories, including movements and directions, in the lung environment under various respiratory patterns. Although our analysis was limited to drug penetration and release through the lung mucosal epithelial layer, the positioning of drug particles from confocal imaging (Figure 5) was effectively utilized to study the behavior of inhaled substances under respiratory patterns. This supports the general applicability of our BMC platform across commonly used in vitro lung models to precisely predict inhaled drug behavior in lung microenvironments.

Upon platform adjustment, our central diffuser and airtight system configuration are crucial for achieving a realistic simulation of aerosol delivery within the microfluidic chip, closely mimicking conditions in the human respiratory system. The real-time microairflow sensing system allows for immediate adjustments and monitoring, ensuring that the airflow remains consistent with physiological conditions. The significant reduction in airflow velocity and stabilization postspeed controller integration highlights the effectiveness of our design in creating a controlled environment for aerosol dynamics study. Furthermore, the ability of our system to generate small, stable respiratory airflows allows for more precise simulations of various respiratory parameters, offering an advantage over traditional transwell^39^ and in vitro exposure models,^40^ which are limited to simulating a single airflow exposure. This advanced functionality underscores the potential of our system to provide a more accurate and versatile platform for studying the impacts of inhaled substances under controlled, replicable conditions, aligning closely with real-life respiratory dynamics. Moreover, unlike traditional lung chip models, which incorporate mechanical stretching of epithelial cells to simulate breathing, our BMC platform exclusively focuses on airflow dynamics. While they simulate both mechanical and airflow aspects of breathing, our platform is specifically designed to study the effects of static and dynamic airflows on mucociliary clearance and drug interactions in the small airway part of the human lung, without involving cellular stretching. This specialized focus allows for a detailed analysis of airflow impacts in isolation, providing unique contributions to the field of inhaled drug delivery.

Moreover, the dynamic 3D simulations performed validate our BMC platform’s capability to replicate and study complex respiratory patterns relevant to human physiology. According to fluid dynamics studies, such as those by Park et al., and computer simulations by Stéphano et al., shear stress in human small airways is typically below 1 pascal.^16,19^ Our findings align with these studies, affirming that our system accurately mirrors human pulmonary conditions. The consistency of shear stress within the physiological range under both low and high shear conditions underscores the precision of our BMC platform in simulating human respiratory mechanics. It is crucial to recognize the simplifications in our COMSOL simulations when comparing them to real human respiratory mechanics. Unlike our model, which assumes laminar flow and constant properties, actual respiratory airflow exhibits transient, turbulent behaviors, especially near bifurcations and in the deeper alveoli. Additionally, our model’s simplified geometry does not account for the anatomical heterogeneity of lung tissue or the effects of humidity and temperature gradients, which significantly impact airflow dynamics and particle behavior. Despite these limitations, our study primarily explores the impact of air shear stress changes within the microchannel on the epithelial cell surface. Our findings indicate that the BMC platform not only replicates the shear stress experienced during human breathing but also demonstrates that variations in the size and frequency of respiratory airflow can lead to corresponding shifts in shear stress within the chip. These insights underscore the model’s relevance in simulating certain dynamic aspects of pulmonary function, even with its inherent simplifications.

Another significant finding of our platform is that increased deposition rates were observed under HSS conditions, consistent with previous research demonstrating that turbulence in areas like nasal pockets and bronchial bifurcations enhances aerosol impaction and deposition.^25^ Microfluidic studies support this finding, showing that increased flow rates elevate the Reynolds number, leading to greater fluid turbulence and deposition rates, a behavior also observed in human airways.^24^ The design of our chip, featuring curved channels at the front and back, mirrors these anatomical structures and enhances particle wall impaction in these regions. Furthermore, the distinctive deposition patterns observed at higher aerosol concentrations, predominantly at the chip’s posterior end, are influenced by increased aerosol exposure variability (Figure S2). Although these patterns at higher concentrations are intriguing, they were not further explored in this study, as such high concentrations are not typically relevant to normal human respiratory conditions and were not the primary focus of our research. Our results also revealed that particle fluorescence intensity increased with each respiratory cycle under HSS conditions, eventually stabilizing, which aligns with fluid dynamics simulations predicting particle deposition during respiratory cycles.^41^ This consistency underscores the effectiveness of our BMC platform in simulating realistic respiratory dynamics, crucial for studying inhaled therapeutics. It highlights the significant impact of respiratory patterns on the distribution and deposition of substances within the airway mucus environment, emphasizing the need to integrate these dynamics into inhaled drug delivery studies to enhance therapeutic efficacy and predictability.

Beyond the characterization of the epithelial barrier and mucus layer on chips, we validated the importance of airflow for MCC by simulating the impact of different breathing modes on particle removal from the mucus layer. The results suggest that minor airflows, which simulate normal breathing patterns, can significantly influence mucus clearance, even when ciliary activity is compromised. This aligns with previous research indicating that airflow can become the primary mucus clearance mechanism when cilia are damaged.^31^ Larger airflows, which disrupt the mucus layer and alter particle movement trajectories, highlight the potential of using controlled airflow to manipulate mucus clearance in therapeutic applications, particularly for conditions like chronic obstructive pulmonary disease, where mucus overproduction is common. The stability of ciliary beat frequency across different airflow exposures suggests that while airflow alters particle movement and mucus clearance, it does not necessarily affect the mechanical function of cilia, provided the mucus layer’s integrity is maintained. This contradicts previous studies’ assumptions,^42^ reinforcing the idea that airflow, rather than solely ciliary action, plays a crucial role in effective mucus clearance. Notably, larger airflows caused a significant shift in particle movement, likely disrupting the mucus layer for substantial particle advancement, similar to cough-induced clearance.^32^ Although this finding contradicts the simulated assumptions of other researchers, it reinforces the notion that airflow is a crucial factor in mucus clearance, consistent with their hypotheses.^31^

The selection of liposomes for this study highlights the significance of nanocarrier design in enhancing pulmonary drug delivery. We observed distinct behaviors between hydrophilic and hydrophobic liposomes, illustrating how surface characteristics influence interactions with lung mucosal barriers. Hydrophilic nanoparticles, hindered by interactions with the mucous layer,^43^ exhibit reduced penetration toward epithelial cells. In contrast, hydrophobic nanoparticles, which naturally avoid aqueous environments,^44^ demonstrate deeper penetration into epithelial layers. This tendency is particularly pronounced under high shear stress conditions that simulate deep inhalation or coughing, potentially enhancing the efficacy of hydrophobic drug formulations.^45^ The confocal imaging studies, shown in Figure 5B,C, further elucidate the effects of nanoparticle surface properties on penetration through the lung’s mucosal barrier. Hydrophilic FluoSpheres, which carry negatively charged carboxylate groups, show limited penetration due to electrostatic repulsion from the similarly charged mucus layers.^46^ Conversely, neutral and hydrophobic DiI liposomes penetrate more effectively, especially under high shear stress, suggesting that shear forces significantly facilitate nanoparticle movement through the mucus.

The hydrophobic liposomes used in this study were supplied by the Taiwan Liposome Company (TLC) and labeled with DiI (1,1′-dioctadecyl-3,3,3′,3′-tetramethylindocarbocyanine perchlorate) for fluorescent tracking. DiI is a lipophilic dye that integrates into the lipid bilayer without significantly changing the liposomes’ surface properties.^47^ The hydrophobic nature of these liposomes comes mainly from their lipid composition, not the DiI labeling. This labeling method allowed us to monitor liposome behavior while preserving their original surface characteristics. Our experimental setup included both hydrophilic nanoparticles (carboxylate-modified FluoSpheres) and hydrophobic ones (DiI-labeled liposomes) to compare how different types of nanoparticles interact in our model system. By using this approach, we aimed to simulate a range of potential nanomedicine formulations with varying surface properties, providing insights into their interactions with the lung’s mucosal barrier under different shear stress conditions.

While our current study offers valuable insights into the behavior of liposomes under various shear stress conditions. However, we recognize that other important factors also impact nanocarrier performance. Variables like zeta potential, membrane fluidity, and PEG modification are known to play key roles in how nanocarriers interact within biological systems.^48^ Due to confidentiality agreements and technical limitations from our collaboration with the TLC, we were not able to fully investigate these factors in this study. Nonetheless, previous research by TLC and other studies has shown that zeta potential influences nanoparticle stability and cell uptake, membrane fluidity affects drug release rates, and PEG modification improves mucus penetration and prolongs circulation time.^35,49^ These observations underscore the importance of tailoring nanocarrier surface properties to optimize drug delivery systems for respiratory diseases, especially under the dynamic shear stresses experienced during both normal breathing and pathological states. These findings offer valuable insights for designing more effective nanocarrier-based therapies for chronic lung diseases, taking into account the physical forces prevalent within the lung microenvironment.

Moreover, our results highlight the influence of dynamic airflow conditions on drug delivery. In the dynamic chip setup (chip with airflow), penetration rates of calcein from liposomes were notably lower than in static conditions, likely hindered by increased shear stress. This observation aligns with higher drug release rates under dynamic conditions, which can be attributed to several mechanisms induced by mechanical stress from airflow. Studies have shown that dynamic shear stress can temporarily deform liposomes, leading to brief pore openings and increased membrane permeability. This effect is especially noticeable in fluid lipid bilayers, where shear forces can rearrange the lipid structure, resulting in defects or localized fluid regions that serve as channels for drug release.^50^ In addition, research indicates that high shear rates boost the fluidity of lipid membranes, altering the organization of lipid chains and making it easier for substances to pass through the bilayer. This aligns with our findings, which suggest that increased membrane fluidity, along with the possible activation of mechanosensitive channels or changes in membrane protein structures, creates more pathways for drug release.^51^ Lastly, similar to previous research, our results show that liposomes subjected to repeated cycles of dynamic stress become more permeable over time. Dynamic flow conditions can also change the interfacial tension between the liposome surface and its surrounding environment, which affects membrane stability and permeability.^52^ The marked differences in peak concentrations and release dynamics between transwell and dynamic chip setups demonstrate the challenges of simulating in vivo conditions. Furthermore, the prolonged release rate of calcein in the chip under airflow suggests that dynamic airflow may affect the sustained availability and efficacy of inhaled drugs. These insights are vital for advancing drug delivery systems in respiratory therapies, underscoring the necessity of accounting for both static and dynamic factors in developing inhaled treatments.

In related animal studies, the plasma half-life (*t*_1/2_) of inhaled liposomes was approximately 55.2 h.^35^ Our in vitro tests, designed to align with these timings, faced challenges in simulating release kinetics beyond 4 h due to the lack of liver metabolism and kidney excretion functions. The chip model showed a sustained-release pattern, with calcein concentrations in the culture medium rising over 24 h, contrasting with the decreasing trends observed in vivo.^35^ Consequently, our study could not accurately compare in vitro and in vivo drug *t*_1/2_ using existing dissolution models. Despite this limitation, our system’s use of airflow shear stress offers a novel approach to in vitro testing of inhaled drug release. In Figure 5E, the transwell group had a significantly higher standard deviation compared to the other groups. The chip w/o airflow, with a nonlinear AUC pattern (*R*^2^ of 0.9579), did not fit the noncompartmental model.^37^ However, the chip w/airflow, with an *R*^2^ of 0.9787 and more linear value distribution, emerged as a promising tool for predicting in vivo drug absorption kinetics. The differences between our calcein in vitro release experiments and animal trials might stem from the BMC platform’s sole use of epithelial cells without endothelial cells or macrophages, potentially leading to overestimated drug penetration rates.^53^ Furthermore, calcein, classified as BCS III, is water-soluble but has limited cell membrane permeability.^54^ In our system, calcein’s penetration into lower layers depends on passive diffusion through the PET membrane or cellular absorption and release, with the latter being the rate-limiting step. This factor, challenging to replicate in traditional in vitro release (IVR) models lacking cellular components, gives our system a distinct advantage. While there are strong IVIVC correlations, discrepancies remain, especially in the chip w/o airflow, which showed a nonlinear AUC pattern (*R*^2^ = 0.9579) and could not be aligned using a noncompartmental model. In contrast, the chip w/airflow, with an *R*^2^ of 0.9787, demonstrated linear value distribution, suggesting its potential as a predictive tool for in vivo drug absorption kinetics. The BMC platform’s restriction to epithelial cells, excluding endothelial cells or macrophages, might lead to overestimations of drug penetration rates.^53^ This, combined with the use of calcein, a BCS III compound, which is water-soluble but has limited cell membrane permeability,^54^ presents challenges. Calcein’s penetration into lower layers in our model relies on passive diffusion or cellular uptake and release, a rate-limiting step not typically represented in traditional IVR models. This highlights our system’s unique advantage in simulating in vivo-like conditions, contributing significantly to drug delivery research and predictive model development.

Finally, our BMC platform currently lacks real-time dynamic imaging of the vertical position of drug particles, so the use of static time points in this study is a limitation. With technological advancements, we aim to enable real-time monitoring of drug pathways on the mucus-cell layer, both horizontally and vertically, to better understand the behavior of inhaled nanomedicines. On the other hand, in our in vitro system designed to simulate animal studies, our model faced significant challenges in mimicking release kinetics beyond 4 h due to the lack of liver metabolism and kidney excretion functions, integral to in vivo processes. The chip model demonstrated a sustained-release pattern, continuously increasing the calcein concentration in the culture medium over a 24 h period. From a future perspective, integrating respiratory patterns and mucosal barrier interactions into our platform will allow for more effective studies on the delivery of inhaled drugs across lung mucosal barriers. By establishing a novel IVR model, we aim to simulate the enhanced functionality of the mucosal barrier and the mechanical stresses of respiratory airflow. This approach will increase the biomimicry of the lung microenvironment and improve the predictive accuracy of IVR models.

## Conclusions

In this study, the development of the BMC platform represents an advancement in inhaled nanodrug delivery, addressing the critical need for more accurate simulations of human respiratory dynamics and mucociliary clearance. Our analysis shows that the BMC platform replicates complex airflow patterns and mucosal barriers, providing crucial insights into how nanocarriers such as liposomes interact with lung tissues. We have demonstrated that both static and dynamic shear stress substantially influence the penetration and release rates of these nanocarriers, underscoring the importance of airflow dynamics in optimizing nanodrug delivery systems. The findings underscore the BMC platform’s potential to enhance personalized inhaled therapies by offering a precise and reliable method for evaluating treatment efficacy. The platform’s ability to simulate a wide range of breathing patterns, from healthy individuals to those with severe respiratory conditions, allows for the optimization of drug formulations and delivery methods for specific patient groups or even individual patients. This customization is particularly relevant for conditions such as cystic fibrosis or COPD, where mucus composition changes significantly affect drug release and penetration. The BMC platform’s capacity to evaluate drug penetration through simulated mucus layers under specific shear stress conditions is crucial for developing more effective mucolytic agents or drug delivery systems designed to overcome the mucus barrier in these conditions.^38^ In addition, the BMC platform realistically simulates inhaled nanodrug delivery conditions and the physiological responses of the respiratory system under various conditions, demonstrating substantial potential as an in vitro drug release evaluation tool. By potentially developing artificial mucus tailored with specific mucin or surfactant ratios to accurately mimic the viscosity and pH of patient-specific mucus, the platform offers opportunities to enhance predictive accuracy for personalized treatments. Although this study focused on HSAEC, our platform has demonstrated the capability to support multicell type cultures. As shown in our previous work,^14^ the BMC platform can successfully coculture lung epithelial and endothelial cells, highlighting its potential to replicate the complex cellular composition of the lung epithelium. This feature is essential for future efforts to develop advanced in vitro models that incorporate diverse cell types, providing a more accurate representation of the lung microenvironment. These advancements could lead to more sophisticated in vitro models that integrate biological and mechanical factors affecting nanodrug delivery and disease progression in the lungs, resulting in more precise drug release models aligned with relevant respiratory airflow patterns.

## Methods

### Manufacture of Chip Devices

In our previous work,^14^ we designed the chip device using SolidWorks software (Dassault Systemes SA, France) and manufactured it from polycarbonate (PC) using injection molding by BIG BRIGHT Machinery Precision CO., Ltd. A key feature of this device is the porous polyethylene terephthalate (PET) membrane (0.4 μm pore diameter) separating the top and bottom channels, essential for creating the tissue interface. We have established a standardized bonding protocol to ensure consistent chip fabrication. For solvent bonding, we treated the PC sheets and the PET membrane with oxygen plasma (60 W, 500 mTorr) for 2 min. These components were then immersed in a 3% (v/v) 95% ethanol solution of (3-glycidyloxypropyl) trimethoxysilane (Glymo) (Sigma, USA) for 1 h and a 3% (v/v) isopropanol (IPA) solution of (3-aminopropyl) triethoxysilane (APTES) (Sigma, USA) for 30 min, sequentially. After rinsing the PET membrane with 100% IPA and drying at 80 °C for 30 min, we assembled it between the top and bottom channels. The bonding process was finalized by applying a compressive force of 0.9 N/m at 80 °C to the assembled chip for 40 min. To ensure structural integrity and usability, each chip undergoes a thorough leakage test after fabrication. This quality control measure is crucial for maintaining the reliability and consistency of our BMC platform across multiple experiments.

### Design of Microphysiological Inhalation System

Our aerosol exposure system, designed to mimic the human microphysiological environment within the chip device, included several key components. We used a programmable commercial diaphragm air pump (SCIREQ) for regulating vital breathing parameters like respiratory rate and volume. Additional elements included a straight-speed controller (Airtac, PSA6D), a one-way valve (PISCO, CVPU4-4), and a central diffuser integrated with a nebulizer (Aeroneb Lab, ANP-1100), all interconnected by medical tubes (Tygon, ADF00002). Airflow, drawn from the incubator and driven by the diaphragm pump, was conditioned to maintain the relative humidity levels typical of lower respiratory tracts, close to 100%. While our study does not model the complete humidity range, this method offers a more realistic simulation of lung conditions compared to previous models. Further details on the specific humidity levels and their measurements will be provided to elucidate the environmental conditions within our experiments. Additionally, the straight-speed controller was crucial for limiting the airflow to match physiological respiratory conditions, and the check valve ensured unidirectional airflow to the aerosol diffuser. This diffuser, structured as a single chamber with a bottom opening, effectively delivered aerosols in a small airflow system. The air pump’s operation was managed by flexWare 8 software (SCIREQ), which allows for the customization of various parameters. For this study, however, we focused on a clinically relevant range.

### Simulation of Airflow and Aerosol Transportation

We conducted a computational fluid dynamics (CFD) simulation in COMSOL Multiphysics (version 5.6, COMSOL AB, Sweden) to evaluate airflow-induced shear stress on human small airway epithelial cells (HSAEC) and aerosol transportation within the chip device. The 3D model of the top channel was initially designed in SolidWorks and then imported into COMSOL. The upper channel was discretized with 82,339 tetrahedra and 11,318 triangular mesh cells. Concurrently, the entire pipeline of the chip device was assumed to be filled with compressible air at 37 °C.

Considering the estimated low Reynolds numbers (*Re* < 100), we assumed laminar flow for the airflow. To replicate the experimental aerosol exposure setup, a time-dependent laminar solver was selected to simulate the constant airflow. The boundary conditions were set to be no slip. The momentum equation, Navier–Stokes (eq 2.1), is employed
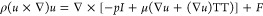
2.1
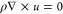
2.2In these equations, *u* represents the velocity vector (m/s), *p* is the pressure (Pa), ρ is the fluid density (kg/m^3^), μ is the coefficient of kinetic viscosity of the fluid (cP), *I* is the unit matrix, and *F* is the volumetric force vector (N/m^3^). Here, the fluid density of the air at 37 °C is 1.225 kg/m^3^, and the coefficient of kinetic viscosity is 0.1893 cP.

As for the regular and rapid breathing patterns, time-dependent solvers were chosen to simulate non-negative sine-wave airflows with angular frequencies of 0.4π and π rad/s, respectively. To match the flow rate in the small airways of the human body, the average velocity of the constant flow or the peak velocities of the sine-wave flow were set to be 0.03317 and 0.005183 m/s. The outlet was set to a zero-pressure condition. A no-slip condition was also applied to the wall. Particles colliding with the walls were only allowed to be deposited on the bottom boundary of the channel. The channel bottom’s shear stress (τ) was calculated by the formula below^16^
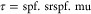
where spf.sr represents the strain rate and the spf.mu means the viscosity. In the particle tracing simulation, 3.5 μm diameter droplets (particle density ρ_p_ = 1000 kg/m^3^) were used to simulate the particle transportation and deposition. To align with our experimental setting, particles were released every 30 s, with 1000 particles released each time. The total amount of particles deposited on the bottom boundary of the channel was evaluated to represent particle deposition within a 15 min duration.

### Measurement of Real-Time Flow

We integrated a microelectromechanical airflow sensor (Honeywell, HAFBSF0200) into our chip device to precisely monitor flow rates into the top channel. This sensor was controlled by an Arduino Uno microcontroller board, which also enabled data communication with an external laptop. The airflow data captured by the sensor were transmitted to and analyzed in Excel (Microsoft) to determine the mean flow rate, along with its peak and minimum values. If discrepancies arose between the calculated flow rate and our target, adjustments were manually made using the direct speed controller. For calibration of the airflow sensor, we used a syringe pump (KD Scientific, Legato 270P) capable of providing known airflow rates. This calibration and monitoring system helps our platform deliver consistent and reliable performance over long durations and across various experiments. The integrated sensor enables real-time adjustments, which improves the reproducibility of our experimental setups.

### Rheology of Chip Membrane Coatings

To simulate a microphysiology-like environment for aerosol transportation in the top channel, we prepared artificial mucus (AM) following Joyner et al.’s method.^55^ We added 4% (w/v) MUC5B (499643, Sigma-Aldrich) and 4% (w/v) MUC5AC (M2378, Sigma-Aldrich) separately to diH_2_O, stirring each for 2 h. These solutions were then polymerized with a cross-linking agent, 4-arm PEG-thiol (Laysan, 4Arm-PEG-SH-20K), and mixed for an additional 2 h to ensure uniformity. The gelation process was completed over 18 h at room temperature. Postgelation, we immediately conducted a time-sweep viscoelasticity measurement in the logarithmic range using a rheometer (HR-1, TA Instruments). We tested shear rates from 1 to 100 Hz at 37 °C, with 10 points per decade.

### Exposure Efficiency of Aerosol Deposition

To observe aerosol transport and deposition within the chip, we prepared a starting solution of either 1 mL of 200 μg/mL fluorescein sodium salt (FSS) or a 0.04% (w/v) suspension of 100 nm carboxylate-polystyrene (PS) particles (580/605) (F8801, Invitrogen). This solution was placed in the nebulizer’s (Aeroneb Lab, ANP-1100) reservoir for aerosolization into the chip’s top channel over 15 min, using a cycle time of 1000 ms and a duty cycle of 5%. Postexposure, the chip was detached, and 100 μL of DI water was introduced into the upper channel to collect the deposited aerosol. This solution was left to dissolve for 3 to 5 min, and its fluorescence intensity was then measured using an ELISA microplate reader (Awareness Technology, ChroMate 4300). A standard curve correlating sodium fluorescein concentration in water with fluorescence intensity was established using Prism 9 (GraphPad). The total aerosol amount deposited in the solution was then deduced using these standard curves.

### Visualization of Aerosol Deposition

Once the aerosol was deposited in the top channel, we placed the chip device in a custom carrier within a high-content system (HCS, Molecular Devices). Using the Molecular Devices ImageXpress Micro4, we captured images of the entire channel. These images were then seamlessly stitched together using MetaXpress software (Molecular Devices), enabling a comprehensive assessment of aerosol distribution within the top channel.

### Model of Human Lung Airway-On-A-Chip

Primary human small airway epithelial cells (HSAEC) (ATCC, PCS-301-010) were cultured in T75 flasks using PneumaCult Ex Plus medium (StemCell, 05040) until 80–90% confluence was reached. Cells at passages 5 to 7 were used for all experiments. An extracellular matrix (ECM) coating was applied to the top channel to promote cell adhesion on the porous PET membrane’s surface. This involved rinsing both channels with 75% ethanol, followed by DPBS (Corning, 21-031-CM) washes. We then filled the top channel with a 300 μg/mL type-I rat tail collagen solution and left the chips to solidify overnight at 4 °C. The HSAEC were trypsinized and then seeded at a concentration of 2.5 × 10^6^ cells/mL with fresh medium, followed by incubation at 37 °C with 5% CO_2_ for 4–6 h under static conditions to boost cell attachment. The apical medium was replenished daily to remove unattached cells. Cultures remained submerged until reaching full confluence, verified by microscopic imaging. Chips not meeting this standard within 5 days were discarded. Approximately 3–5 days postseeding, we established an air–liquid interface (ALI) to stimulate mucociliary differentiation, switching the medium from EX Plus to ALI medium (StemCell, 05001). Morphology, cilia beating, and mucus secretion were regularly checked to assess cell differentiation and function. Over 4 weeks, the apical surface was washed weekly with DPBS to clear debris and mucus. The bottom channels received a continuous 120 μL/h medium flow at 37 °C and 5% CO_2_. The chip devices were checked every 3 days for flow issues and cellular morphology. Any flow disruption in the bottom channel necessitated disconnection from the pump, rinsing with fresh medium to clear air bubbles, and reconnection.

### Mucus Quantification

After the establishment of the air–liquid interface (ALI), an examination was carried out on the mucus produced by the HSAEC. The method of mucus extraction entailed the introduction of 50 μL of prewarmed DPBS into the upper channel, followed by an incubation at 37 °C for 10 min. Subsequently, the resultant sample solution was gathered and preserved at −20 °C for future evaluation. To measure the quantity of mucus generated, a spectrophotometer (Implen, NP80) was employed in combination with a sulfated-Glycosaminoglycans Assay Kit (Abcam, ab289846). Additionally, to quantify the thickness of mucus within the microchannels, the mucus was stained with MUC5B and measured directly under a confocal microscope. The thickness was determined by averaging measurements from five randomly selected fields of view within the channel, ensuring a consistent and representative assessment of the mucus layer’s dimensions.

### Ciliary Beat Frequency Measurement

To measure the ciliary beat frequency, we observed the HSAEC with differentiated cilia after 3 weeks of air–liquid interface culture. Using an inverted microscope system (Nikon, Eclipse Ti), we recorded bright-field videos of ciliated surfaces with a high-speed camera (SagaView, SP150) at 100 fps and a 1440 × 1080 acquisition matrix. We captured videos from more than five fields of view per chip. Cilia activities were automatically detected using a previously described method.^33^ This involved a MATLAB (MathWorks) script calculating the time-dependent grayscale changes of each pixel across frames. It identified the periodicities of brightness change, corresponding to ciliary movement, and applied a threshold. We then used a bandpass filter ranging from 1 to 30 Hz to minimize noise. The final step was to map the ciliary beat frequency for each field of view and average these values for statistical analysis.

### Quantification of Airway Mucociliary Clearance (MCC)

To observe the mucociliary clearance, a dilution of polystyrene (PS) red fluorescent particles (FluoSpheresTM, Carboxylate-modified microspheres, 2 μm (580/605), 2% solids, F8826, Thermo Fisher Scientific) in DPBS with a concentration of 40 μg/mL was aerosolized and subsequently delivered to the apical surface of the epithelium as previously described. The movement of the particles was then recorded by an inverted fluorescence microscope (magnification of 20×) for 1–5 min at 30 fps with a 536 × 536 pixel resolution. A custom Python script was then employed to identify fluorescent signals at each time step and chart the movement of all particles within the field of view. This allowed us to automatically quantify the trajectories, and thereby estimate both the velocity and direction of MCC.

### Immunofluorescence Staining

After removing the culture medium, both channels were rinsed with prewarmed DPBS, followed by fixing the cells with 4% paraformaldehyde (PFA)/for 10–15 min at room temperature. To visualize mucous tissue thickness, we applied 10% (v/v) acetic acid directly to the mucus in the apical channel for 2 h, skipping the preliminary DPBS wash. Postfixation, the channels underwent three DPBS rinses. A blocking buffer of 1% BSA and 5% FBS in DPBS was then introduced to minimize nonspecific binding, resting for 30 min at room temperature. Primary antibodies, as specified in the Supporting Information table and diluted in DPBS at ratio of 1:200, were then applied to the fixed cells for overnight incubation at 4 °C. This was followed by two DPBS washes and a subsequent 2 h incubation with the corresponding secondary antibodies at room temperature, as outlined in the Supporting Information table. The membrane was carefully removed as part of disassembling the chip and immersed in a DAPI-containing mounting medium (VECTASHIELD, H-1200) for nuclear staining. The final step involved fluorescence imaging using a Leica TCS SP8 confocal laser-scanning microscope, with image processing conducted in Adobe Illustrator software.

### Analysis of Liposome Penetration

To assess shear stress effects on particle penetration into the mucus layer, DiI-labeled liposomes (Taiwan Liposome Company) were aerosolized onto HSAEC’s apical surface using the established aerosol exposure method. The samples underwent fixation and immunofluorescence staining. Three-dimensional images of the HSAEC were captured at 40× magnification using the confocal microscope. The side view of the three-dimensional (3D) image determined the liposome’s *Z*-axis penetration depth and position. Subsequently, the 3D cell images were analyzed using custom MATLAB code. The DAPI fluorescence signal identified the upper and lower boundaries of the entire mucosal barrier, including both the mucus and cell layers. This total barrier thickness was then segmented into three equal divisions along the *Z*-axis, regardless of the absolute mucus thickness. This standardized segmentation method allows for consistent analysis across different shear stress conditions, with the upper section primarily representing the mucus layer, the middle section representing the mucus-cell interface, and the lower section primarily representing the cellular layer. To conclude the analysis, we quantified the proportion of red fluorescent signals (representing DiI-liposomes) in each division relative to the total red signals, providing insights into the relative distribution of the liposomes throughout the mucosal barrier under different shear stress conditions.

### In Vitro Release Analysis

Before aerosol exposure, the small airway chip was detached from the peristaltic pump, maintaining a static lower channel. We added 200 μL of fresh medium to ensure cell viability. In parallel, the transwell’s lower chamber was replenished with 700 μL of fresh medium. During the experiment, the small airway chip’s top channel was subjected to a 100 mL/min airflow for 3 min to deposit liposomes in the flow channel. The transwell, positioned under the nebulizer, allowed for liposome sedimentation onto the mucus. Postdeposition, chips with airflow were exposed to 50 mL/min biomimetic respiratory airflow and incubated at 37 °C/5% CO_2_ incubator. Chips without airflow and the transwells were kept static under similar incubation conditions. At six intervals (15 min, 30 min, 1, 2, 4, and 24 h postdeposition), we retrieved and replaced 200 μL of culture medium from each chip’s lower layer. After 24 h, we simultaneously removed the mucus and cell layers. For mucus extraction, cells were treated with a 6.5 mM DTT solution and incubated at 37 °C for 30 min. Post-DTT aspiration, cells were washed twice with DPBS. Then, 1% Triton X-100 was applied to the cell layer, incubated for 10 min, followed by two DPBS washes to finalize cellular fluid extraction. All samples were stored at −20 °C for later analysis. DiI liposome and calcein concentrations in these samples were quantified using a microplate reader (CLARIOstar, BMG Labtech). We dispensed 100 μL from each sample into a 96-well plate (165305, Nunc) and set the reader for fluorescence: λ_ex_: 548/8 nm, λ_em_: 567/8 nm for DiI; λ_ex_: 493/8 nm, λ_em_: 527/8 nm for calcein. The fluorescence intensity was recorded and converted to relative concentration (%) using a standard curve (Figure S9).

### Correlation between IVR and In Vivo Absorption Fraction

The in vivo drug absorption data from mouse experiments with HCQ liposomes, as reported by TLC, were used to compare with our in vitro results.^35^ The in vitro release and in vivo concentration levels were calculated using the cumulative area-under-curve (AUC) at 0.25, 1, 4, and 24 h employing a noncompartmental method.^35^ Pearson’s correlation analysis was conducted to determine the *R* squared between in vitro and in vivo results.

### Statistical Analysis

All results presented are from at least 3 independent experiments. The student’s *t*-test or one-way/two-way ANOVA was performed using GraphPad Prism version 6.0 (GraphPad Software Inc.) for statistically evaluating quantified data. Differences between groups were considered statistically significant when *p* < 0.05 (*p < 0.05, **p < 0.01, ***p < 0.001).

## Supporting Information Available

Additional data include simulations of shear stress and velocity changes within the system, fluorescent imaging showing particle deposition and cellular growth over a 21 day period under varied conditions, flow cytometry analysis quantifying nanoparticle size distribution and behavior in the microfluidic setup, assessments of ciliary beat frequency of human airway epithelial cells under different respiratory patterns, investigations of particle distributions across various breathing patterns, studies on deposition rates of liposomes in multiple experimental setups (PDF)
Movie S1: Dynamic visual evidence of particle movements under high shear stress conditions (MOV)

## Supplementary Material

nn4c11693_si_001.pdf

nn4c11693_si_002.mov
