# Supplementary material for: Inhalable Mucociliary-On-Chip System Revealing Pulmonary Clearance Dynamics in Nanodrug Delivery
Source: ACS Nano. 2025 Jan 7;19(2):2228–44. doi: 10.1021/acsnano.4c11693 (PMC11760172; doi:10.1021/acsnano.4c11693)
Supplement: Supplementary file 1 — nn4c11693_si_001.pdf [file nn4c11693_si_001.pdf]

**Supplemental information**

# Inhalable Mucociliary-on-Chip System Revealing Pulmonary Clearance Dynamics in Nanodrug Delivery

Ko-Chih Lin[1,2], Hsuan-Yu Lin[2], Chuan-Yi Yang[1,2], Ying-Ling Chu[1,2], Ren-Hao Xie[1,2], Cheng-Ming Wang[2], Yun-Long Tseng[3], He-Ru Chen[3], Johnson H.Y Chung[4], Jia-Wei Yang[5]* and Guan-Yu Chen[1,2,6,7]*

[1]Department of Electrical and Computer Engineering, National Yang Ming Chiao Tung University, Hsinchu 30010, Taiwan
[2]Institute of Biomedical Engineering, College of Electrical and Computer Engineering, National Yang Ming Chiao Tung University, Hsinchu 30010, Taiwan
[3]Taiwan Liposome Company, Ltd, Taipei 11503, Taiwan
[4]Intelligent polymer research institute, AIIM Facility, University of Wollongong 2500, Australia
[5]Anivance AI Corporation, Hsinchu, Taiwan
[6]Department of Biological Science and Technology, College of Biological Science and Technology, National Yang Ming Chiao Tung University, Hsinchu 30010, Taiwan
[7]Center for Intelligent Drug Systems and Smart Bio-devices (IDS$^2$B), National Yang Ming Chiao Tung University, Hsinchu 30010, Taiwan

*Correspondence: guanyu@nycu.edu.tw (G.Y. C) and jack.yang@anivance.io (J.W. Y)

Phone: (886) 3-573-1920

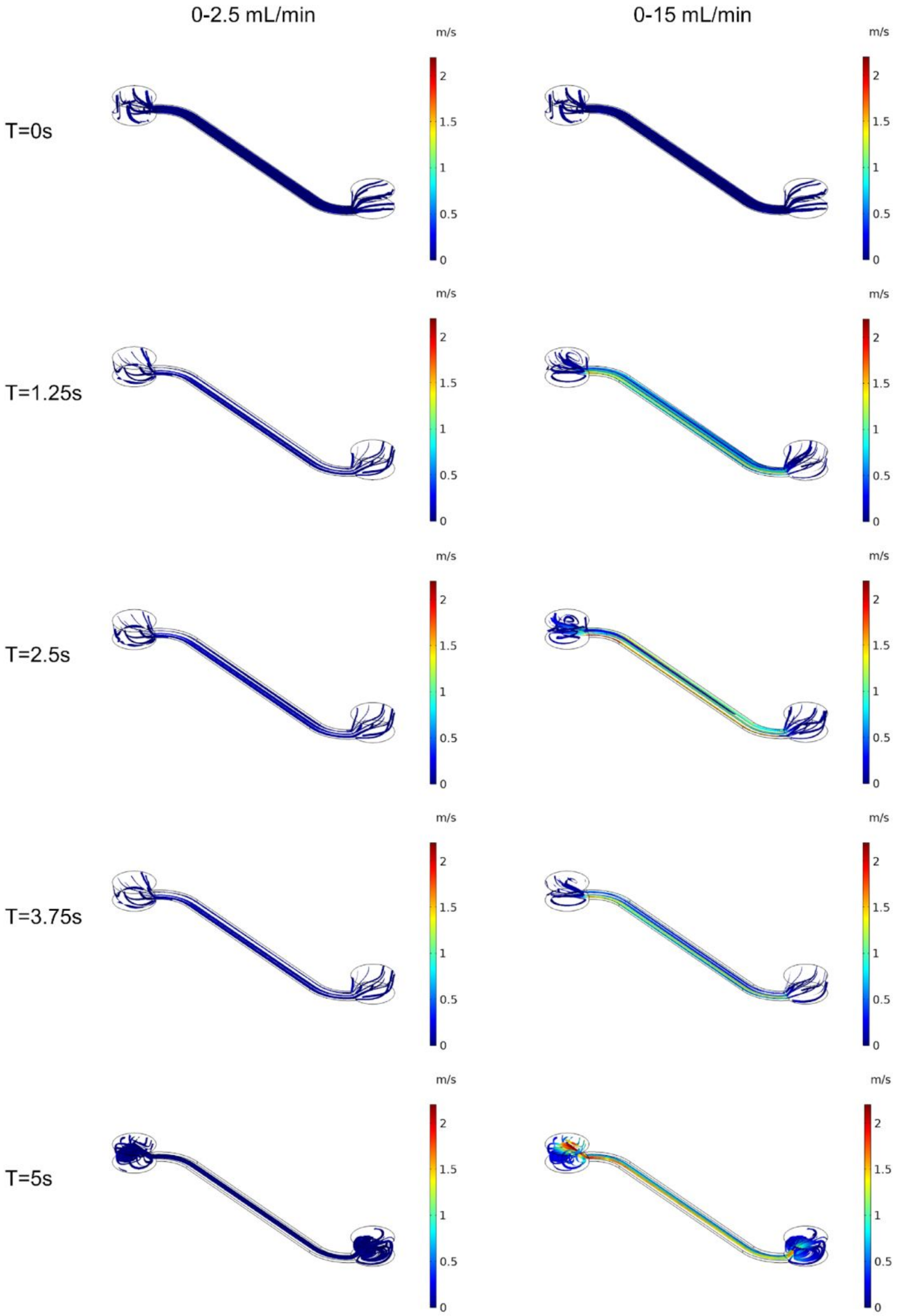


**Figure S1. Simulated shear stress and velocity magnitude in the chips under two flow patterns.** Computational fluid dynamics simulation showing two air-breathing flow velocity profiles (0-2.5 mL/min and 0-15 mL/min) in the microfluidic chip, cycled over a 5-second period. Velocity magnitudes are represented by varying colors.

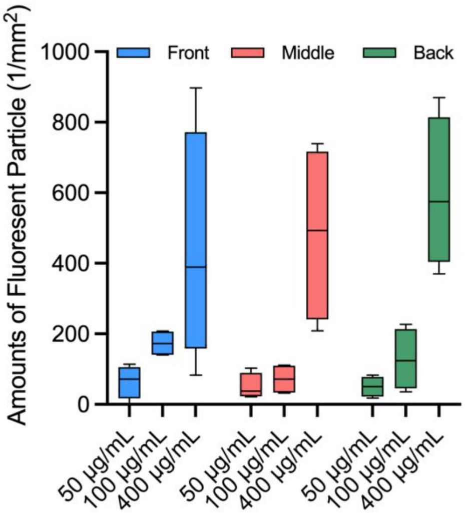


**Figure S2. Fluorescent intensity of particle deposition in the BMC under HSS conditions.** The amounts of fluorescent particles (1/mm²) deposited in the front (blue), middle (red), and back (green) sections of the BMC at different aerosol concentrations (50 µg/mL, 100 µg/mL, and 400 µg/mL).

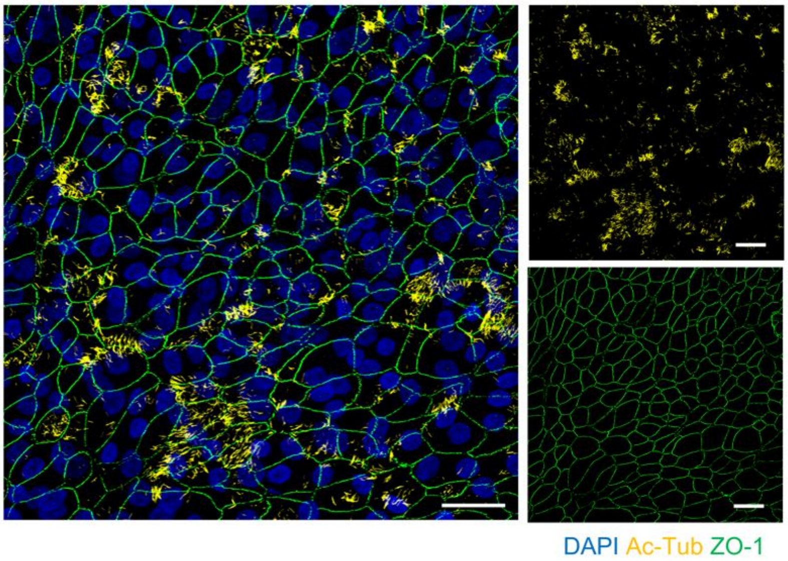


**Figure S3. The fluorescent confocal images of HSAECs cultured on the BMC for 21 days.** The main image shows the cells stained with DAPI (nuclei, blue), Ac-Tub (cilia, yellow), and ZO-1 (tight junctions, green). Insets highlight the distribution of cilia across the cell surface (top right, Ac-Tub) and the network of tight junctions between cells (bottom right, ZO-1). Scale bars: 25 µm.

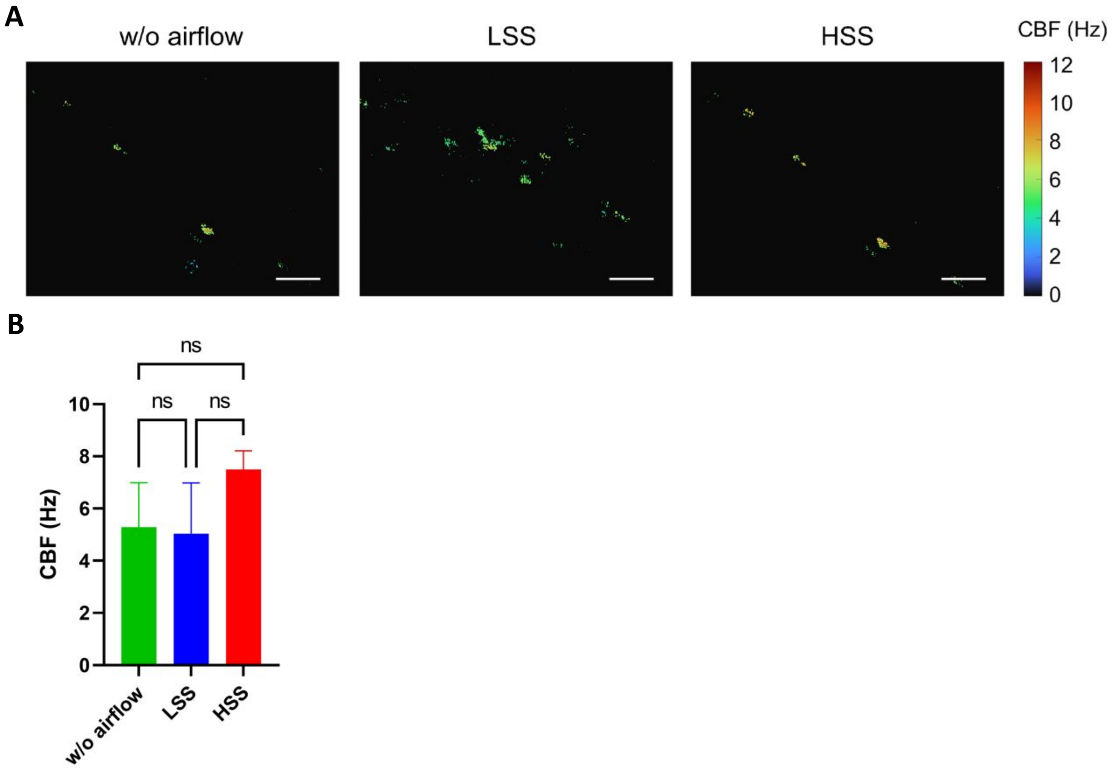


**Figure S4. The CBF assessment of HSAECs under various respiratory patterns in the BMC platform.** (A) The heatmaps illustrate the CBF under three conditions: w/o airflow, LSS, and HSS. The intensity of CBF activity is indicated by varying color gradients, ranging from no activity (black, 0 Hz) to the highest activity (red, 12 Hz). Scale bars: 100 µm. (B) Bar graph displaying comparative CBF across varying respiratory conditions. 'ns' denotes a non-significant difference between the groups.

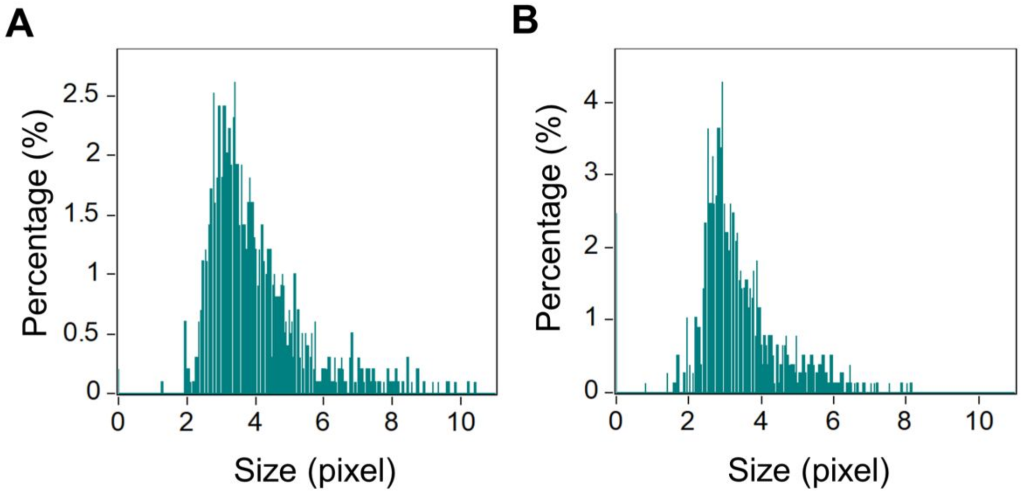


**Figure S5. Size distribution of 100 nm PS particle and Dil-liposomes by flow cytometry analysis.** (A) The majority pixel sizes of 100 nm PS particle range between 2 and 6 pixels, with a peak at approximately 3 pixels. (B) The majority pixel sizes of Dil-liposomes are concentrated between 2 and 6 pixels, with a peak at around 3 pixels.

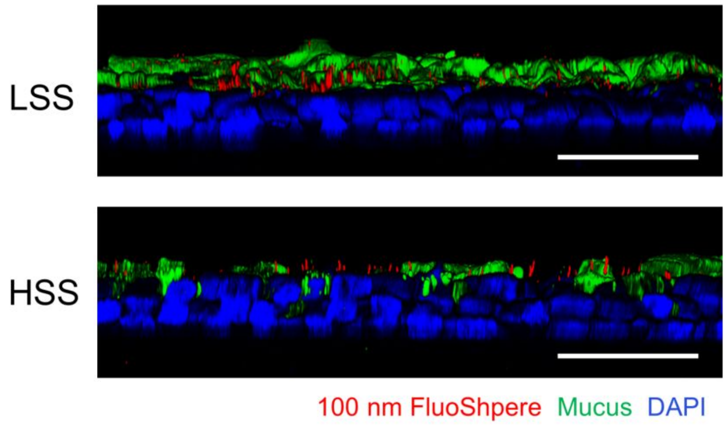


**Figure S6. The distribution of 100 nm FluoSphere particles in the mucus layer under different breathing patterns.** The confocal microscopy images of HSAECs cultured on the BMC and stained for MUC5B (mucus) and with DAPI (nuclei), exposed to LSS and HSS. Fluorescent FluoSpheres are marked in red, the mucus layer in green, and cell nuclei in blue. Scale bar: 50 µm.

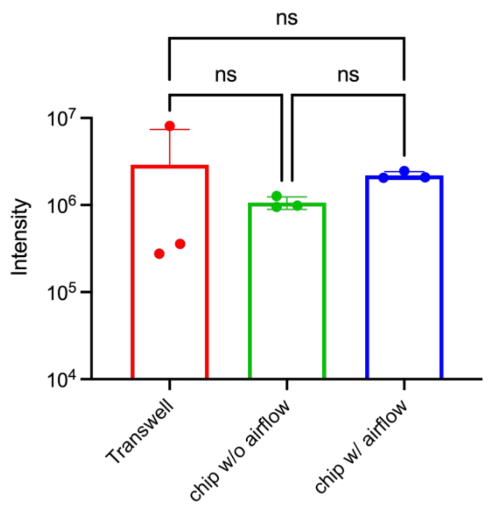


**Figure S7. Validation of the differences in DiI-liposome deposition rates across different setups during initial exposure.** Fluorescence intensity measurements of DiI-liposome in the mucus layer for transwell, chip without airflow, and chip with airflow conditions. Statistics were performed using 1-way ANOVA with multiple comparisons. The bar graph indicates no significant differences ('ns') between any of the conditions.

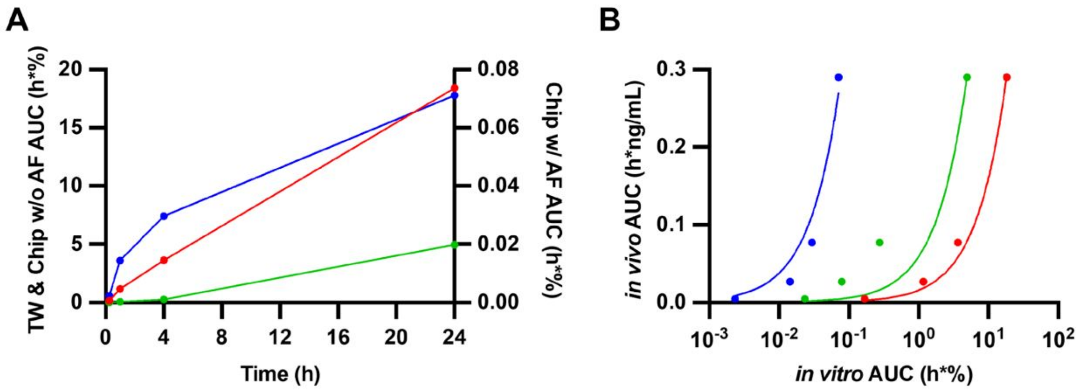


**Figure S8. Evaluation of BMC platform's predictive ability using in *vitro-in vivo* correlation (IVIVC).** (A) Area-under-the-curve (AUC) vs. time plots for DiI-calcein liposome release under different setups: transwell (red), chip w/o AF (green), and chip w/ AF (blue). The plot illustrates the cumulative calcein release over 24 hours, highlighting the differences in release dynamics among the setups. (B) Correlation between *in vitro* AUC and *in vivo* AUC for transwell (red), chip w/o AF (green), and chip w/ AF (blue). The plot shows the predictive accuracy of the *in vitro* models in relation to *in vivo* results.

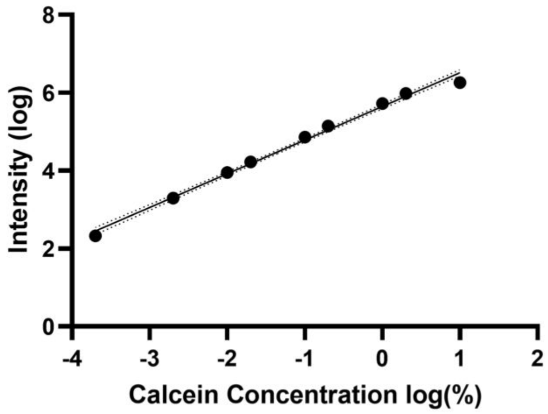


**Figure S9. Calibration curve for calcein concentration quantification.** The fluorescence intensity was recorded and converted to relative calcein concentration (%) using this standard curve, validating the use of fluorescence intensity for quantifying calcein concentration in the BMC platform.

**Table S1.** ***In vitro*** **pharmacokinetic testing parameters for inhaled DiI-calcein liposomes under different setups.**

| PK parameters | Transwell | Chip w/o airflow | Chip w/ airflow |
|---|---|---|---|
| $C_{max}$ (%) | 1.344329 | N/A | 0.018698 |
| $T_{max}$, hours | 0.25 | 0.25 | 0.25 |
| $AUC_{0-24}$, hour*% | 18.41056 | 4.953006 | 0.07116 |

**Movie S1.** Real-time video showing particle trajectories in the BMC under the HSS condition. Under HSS, particles exhibit intense back-and-forth movement, eventually aligning with the airflow direction.
